# ﻿Description of immature stages of *Gymnetron* species (Coleoptera, Curculionidae, Curculioninae), with particular emphasis on the diagnostic morphological characters at the generic and specific levels

**DOI:** 10.3897/zookeys.1090.78741

**Published:** 2022-03-23

**Authors:** Jiří Skuhrovec, Rafał Gosik, Roberto Caldara, Ivo Toševski, Aleksandra Batyra

**Affiliations:** 1 Group Function of Invertebrate and Plant Biodiversity in Agro-Ecosystems, Crop Research Institute, Prague 6–Ruzyně, Czech Republic Group Function of Invertebrate and Plant Biodiversity in Agro-Ecosystems, Crop Research Institute Prague Czech Republic; 2 Department of Zoology and Nature Protection, Institute of Biological Sciences, Maria Curie-Skłodowska University, Akademicka 19, 20–033 Lublin, Poland Maria Curie-Skłodowska University Lublin Poland; 3 Center of Alpine Entomology, University of Milan, Via Celoria 2, 20133 Milan, Italy University of Milan Milan Italy; 4 CABI, Rue des Grillons 1, 2800 Delémont, Switzerland CABI Delémont Switzerland; 5 Institute for Plant Protection and Environment, Banatska 33, 11080 Zemun, Serbia Institute for Plant Protection and Environment Zemun Serbia; 6 Żabia Wola 75H, 23–107 Strzyżewice, Poland unaffiliated Strzyżewice Poland

**Keywords:** Biology, mature larva, Mecinini, morphology, pupa, taxonomy, weevils

## Abstract

The immature stages of the following five Palaearctic *Gymnetron* species are described for the first time: *G.tibiellum* Desbrochers des Loges, 1900, *G.veronicae* (Germar, 1821), *G.rotundicolle* Gyllenhal, 1838, *G.melanarium* (Germar, 1821), and *G.villosulum* Gyllenhal, 1838. These species belong to four different groups previously established according to a phylogenetic analysis: the first two belong to the *G.veronicae* group and the other three to groups respectively bearing their name (*G.rotundicolle*, *G.melanarium*, and *G.villosulum* groups). All these species exhibit several diagnostic characters distinguishing them from each other. Some characters that can be used to separate *Gymnetron* from other genera in the tribe are also suggested. Three highly significant characters for the larvae and three for the pupae were identified. For the larvae they are: (1) labial palpi with single palpomeres, (2) all spiracles unicameral, and (3) epipharynx with a single pair of *mes* or none at all. For the pupae they are: (1) the pronotum with prominent pronotal protuberances, (2) abdominal segment VIII with a conical abdominal protuberance dorsally, and (3) very short or even reduced urogomphi. The species studied here are compared with those *Gymnetron* species already known and with other genera in the tribe Mecinini. Keys to the larvae and pupae described here are provided. All the characters used for identification are illustrated by photographs or drawings.

## ﻿Introduction

The genus *Gymnetron* Schoenherr, 1825 belongs to the tribe Mecinini (Curculionidae, Curculioninae) and includes some 35 Palaearctic species (Caldara 2008a; Alonso-Zarazaga et al. 2017) and 70 Afrotropical species (Caldara 2003). The adults of this tribe were recently subjected to morphological revision and phylogenetic analysis (Caldara 2003, 2008a). Based on this analysis, nine Palaearctic species groups and 13 Afrotropical species groups were recognized. Within this tribe the genus *Gymnetron* seems more closely related to *Rhinusa* Stephens, 1829 than to other genera (Caldara 2001). Preliminary molecular studies appear to confirm this placement (Hernández-Vera et al. 2013; I. Toševski unpublished data).

The Palaearctic *Gymnetron* species live on *Veronica* (Caldara 2008a), currently included in Plantaginaceae (Olmstead et al. 2001; Albach et al. 2004), whereas those in the Afrotropical region (Caldara 2003; Caldara et al. 2010), where Plantaginaceae are poorly represented, appear to live on various genera of Scrophulariaceae distributed mainly in the southern hemisphere, i.e., *Diascia*, *Hemimeris* and *Nemesia* (Hemimerideae), *Hebenstreitia*, *Selago*, *Sutera* and *Tetraselago* (Selagineae), *Buddleja* (Buddlejeae), as well as on Stilbaceae, i.e., *Anastrebe*, a plant genus previously placed within Scrophulariaceae (Olmstead et al. 2001; APG 2016). The larvae develop inside the ovaries, stems or roots of the host plants and can sometimes induce the formation of galls (Hoffmann 1958; Caldara 2001).

To date, larvae and pupae of only three species of *Gymnetron* (*G.auliense* Reitter, 1907, *G.miyoshii* Miyoshi, 1922, and *G.vittipenne* Marseul, 1876) have been adequately described (Jiang and Zhang 2015). Immatures of some other *Gymnetron* species – *G.anagallis* Marshall, 1933 (Gardner 1934; van Emden 1938); *G.beccabungae* (Linnaeus, 1760) and *G.villosulum* Gyllenhal, 1838 (van Emden 1938; Scherf 1964) – have been previously studied, but no detailed descriptions are available.

Therefore, the aims of the present study are to describe larvae and pupae of five *Gymnetron* species in detail for the first time, to find characters that are diagnostic at the generic and specific levels, and finally, to compare the characters of the immature stages of this genus with other genera of the same tribe that might be phylogenetically informative.

## ﻿Materials and methods

The material for this study, i.e., L3 larvae and pupae from each of the species studied was collected from their host plants together with the adult, and subsequently preserved in 2 ml screw-cap micro tubes (Sarstedt, Germany) filled with 96% ethanol at 4–6 °C. The insect taxa were identified by Roberto Caldara, those of the plants by Ivo Toševski.

Part of the larval and pupal material was preserved in glycol or Pampel fixation liquid (see Skuhrovec and Bogusch 2016) and used for the morphological descriptions. These specimens are now deposited in the Group Function of Invertebrate and Plant Biodiversity in Agro-ecosystems of the Crop Research Institute (Prague, Czech Republic). Slide preparation basically followed May (1994). The larvae selected for study under the microscope were cleared in 10% potassium hydroxide (KOH), then rinsed in distilled water and dissected. After clearing, the head, mouthparts and body (thoracic and abdominal segments) were separated and mounted on permanent microscope slides in Faure-Berlese fluid (50 g gum arabic and 45 g chloral hydrate dissolved in 80 g of distilled water and 60 cm^3^ of glycerol) (Hille Ris Lambers 1950).

All the specimens described were fixed in 95% ethanol and examined under an optical stereomicroscope (Olympus SZ 60 and Nikon Eclipse 80i) with calibrated oculars. The following measurements of larval instars were made: body length (BL), body width (BW) (at the third abdominal segment) and width of the head capsule (HW) (see Gosik et al. 2016). The pupal measurements included body length (BL), body width (BW) (at the level of the mid legs), head width (HW) (at the level of the eyes), length of rostrum (RL) and width of pronotum (PW). All the measurements are given in Table 1 (mature larva) and Table 2 (pupa).

**Table 1.** Measurements (in mm) of body parts (mature larva) in the *Gymnetron* species studied here; ^n^ = number of specimens.

Species	Body length	Body width	Head width
*G.melanarium*	2.33^2^, 3.00^1^, 2.66^1^	0.76^1^, 1.00^3^	0.50^2^, 0.53^2^
*G.rotundicolle*	2.20^13^, 2.25^12^, 2.33^5^, 2.26^7^	0.75^28^, 0.86^9^	0.40^14^, 0.47^13^, 0.50^14^
*G.tibiellum*	2.25^1^, 2.37^4^, 2.50^4^	0.87^6^, 0.95^3^	0.42^7^, 0.45^2^
*G.veronicae*	2.16^1^, 2.56^1^, 2.83^1^, 3.00^1^	0.76^1^, 1.00^3^	0.50^2^, 0.53^2^
*G.villosulum*	2.25^1^, 2.33^2^, 2.46^1^	0.83^2^, 1.10^2^	0.40^1^, 0.51^3^

The drawings and outlines were made using a drawing tube (MNR–1) installed on a stereomicroscope (Amplival) and processed by computer software (Corel Photo-Paint X7, Corel Draw X7). The thoracic spiracle was located on the prothorax near the boundary of the prothorax and mesothorax, as shown in the drawing, but this spiracle is of mesothoracic origin (Marvaldi et al. 2002; Marvaldi 2003). The drawings show the thoracic and abdominal spiracles. The lengths of all setae are visible in the figures. The numbers of setae of the bilateral structures are given for one side.

**Table 2.** Measurements (in mm) of body parts (pupa) in the *Gymnetron* species studied here; ^n^ = number of specimens; BL = body length; BW = body width; THW = head width.

Species	Female			Male		
	BL	BW	THW	BL	BW	THW
*G.melanarium*	2.12^2^, 2.32^1^	1.25^3^	0.75^3^	2.25^1^	1.32^1^	0.82^1^
*G.rotundicolle*	2.37^1^, 2.62^3^	1.32^3^, 1.42^1^	0.75^3^, 0.85^1^	2.20^2^, 2.22^1^	1.12^2^, 1.32^1^	0.70^3^
*G.tibiellum*	1.87^1^, 2.25^1^, 2.50^1^	0.62^3^, 0.75^1^	0.75^1^, 1.12^3^	1.92^1^, 2.07^3^	0.92^1^, 1.1^2^, 1.25^1^	0.67^1^, 1.00^3^
*G.veronicae*	2.12^2^, 2.32^1^	1.25^3^	0.70^1^, 0.75^2^	2.25^1^	1.32^1^	0.80^1^
*G.villosulum*	2.24^3^, 2.50^5^, 2.73^3^	1.30^2^, 1.35^4^, 1.50^3^, 1.55^2^	0.82^4^, 0.87^4^, 0.88^3^	2.24^1^, 2.40^3^, 2.60^2^	1.32^2^, 1.35^4^	0.82^3^, 0.87^3^

The terms and abbreviations for the setae of the mature larvae and pupae are as in Scherf (1964), May (1977, 1994) and Marvaldi (1997, 1999), but see also Skuhrovec (2007). The antennae terminology follows Zacharuk (1985).

The sequence of the species follows that proposed by Caldara and Fogato (2013) and Caldara et al. (2013).

The botanical taxonomy follows APG IV (APG 2016).

## ﻿Results

### ﻿Morphology of immature stages

#### ﻿Genus Gymnetron

Taxon classificationAnimaliaColeopteraCurculionidae

Schoenherr, 1825

1210DD17-666A-549D-94C1-E5FD13D82FB1

##### Description of mature larva (L3).

***Measurements*** (in mm). Body length: 2.16–3.00. The widest point in the body (meso- and metathorax) measures up to 1.20. Head width: 0.36–0.53.

***General*.** Body elongate or relatively elongate, slender, weakly curved, rounded in cross section.

***Colouration*.** Pale yellow or dark brown head. All thoracic and abdominal segments white, cuticle smooth or with many reddish or brown asperities.

***Vestiture*.** Setae on body thin, distinctly different in length (minute to very short or long).

***Head capsule*.** Head almost oval or suboval, endocarinal line present. Frontal sutures on head distinct, extended to antennae. One stemma, in the form of a pigmented spot with convex cornea, both located on each side anterolaterally, above frontal suture. Dorsum of epicranium with three or five setae; *des_1_* located in central part of epicranium; *des_2_* lateral, sometimes absent; *des_3_* located anteriorly on epicranium close to frontal suture; *des_4_* often medially, sometimes absent; *des_5_* located anterolaterally. Frons with three to four *fs*, *fs_1_* absent, *fs_2_* located medially, *fs_3_* sometimes absent, *fs_4_* and *fs_5_* subequal. Head with two *les*, one or two *ves*, and two to six *pes*.

***Antennae*** located at end of frontal suture on each side, membranous and distinctly convex basal article bearing one conical sensorium, relatively long.

***Clypeus*** trapezium-shaped, ~ 3–4× as wide as long with two relatively long *cls*, located posterolaterally.

***Mouth parts*.** Labrum ~ 3–4× as wide as long, with three piliform *lms*, relatively long; anterior margin doubly sinuate. Epipharynx with two or three long digitate *als*; with two or three *ams*, and one or without *mes*; labral rods indistinct. Mandibles distinctly broad, bifid, teeth of unequal height; slightly truncate; both *mds* relatively long, piliform, located in distinct holes. Maxilla: stipes with one *stps*, two *pfs* and sensillum, with or without *mbs*; mala with four or five elongated digitate *dms*; three or four *vms*, of various length; all *vms* distinctly shorter than *dms*. Maxillary palpi with two palpomeres; basal palpomere with one *mxps* and one sensillum; distal palpomere with one sensillum and a group of conical, cuticular apical processes. Praelabium oval, with one *prms*; ligula with two *ligs*. Labial palpi with one palpomere; palpomere with one sensillum and short, cuticular apical processes. Postlabium with two or three *pms*, all located laterally; membranous area finely or distinctly asperate.

***Thorax*.** Prothorax distinctly smaller than meso- and metathorax. Spiracle unicameral, situated between pro- and mesothorax (see Material and methods). Prothorax with seven to eleven *prns*; two *ps*; and two *eus*. Mesothorax with or without two *prs*; two or three *pds*; one long *as*; two or three *ss*; one *eps*; one *ps*; and one or two *eus*. Each pedal area of thoracic segments well separated, with three or five *pda*.

***Abdomen*.** Abdominal segments I–III of almost equal length, next abdominal segments shortening gradually to the terminal parts of the body. Abdominal segment X reduced to four anal lobes of unequal size, the lateral lobes being distinctly the largest, and the dorsal and ventral ones very small. Anus located terminally; ambulatory ampullae bilobate to circular. Spiracles unicameral, seven abdominal spiracles located laterally. Abdominal segments I–VI with one or two *prs*; one or two *pds*; two *ss*; one *eps*; one or two *ps*; one *lsts* and one or two *eus*. Abdominal segments VII–VIII without, one or two *prs*; one or two *pds*; one or two *ss*; one *eps*; one or two *ps*; without or one *lsts*; and one or two *eus*. Abdominal segment IX with one or two *ds*; one or two *ps*; and one or two *sts*. Abdominal segment X with one or two setae (*ts*).

##### Description of pupa.

***Measurements*** (in mm). Body length: 1.87–2.73. Body width: 0.62–1.55. Thorax width: 0.67–1.12.

***Body*.** Moderately stout, yellowish or brownish. Pronotal protuberances (p-pr) sclerotized, prominent, body covered with fine, knobby asperities; fused at base or well separated. Rostrum rather or moderately slender, ~ 4× as long as wide, extending to mesocoxae. Antennae rather short, clava smooth. Pronotum 1.5–2.2× as wide as long. Mesonotum slightly or sometimes distinctly smaller than metanotum. Abdominal segments I–V of equal length; segments VI–VIII tapering gradually to the terminal part of the body, segment IX distinctly reduced. Spiracles on abdominal segments I–V functional. Urogomphi reduced or short. Abdominal segment VIII with well visible conical abdominal protuberance dorsally (a-pr), extending the outline of the body.

***Chaetotaxy*.** Sparse, setae of different lengths, transparent. Head with one or two *os*. Rostrum with or without one *rs*. Pronotum with one or two *as*, one or two *ds*, with two or without *sls*, one or three *ls* and three or four *pls*. Dorsal parts of meso- and metathorax with two or three setae. Apex of femora with one or two *fes*. Abdominal segments I–VIII with two or five setae dorsally. Each lateral part of abdominal segments I–VIII with one or two setae. Ventral parts of abdominal segments I–VIII with two or three setae. Abdominal segment IX with two setae ventrally.

### ﻿Descriptions of immature stages of the species

#### ﻿ Gymnetron tibiellum

Taxon classificationAnimaliaColeopteraCurculionidae

Desbrochers des Loges, 1900

3BD385E8-BAA4-502A-AEAD-CC91E2490919

##### Material examined.

Serbia, Bela Palanka, 43°13.150'N, 22°18.886'E, 288 m, ex *Veronicaanagallis-aquatica*, 29.06.2020, leg. Toševski (9 larvae and 9 pupae).

##### Description of mature larva

**Figure 1.**
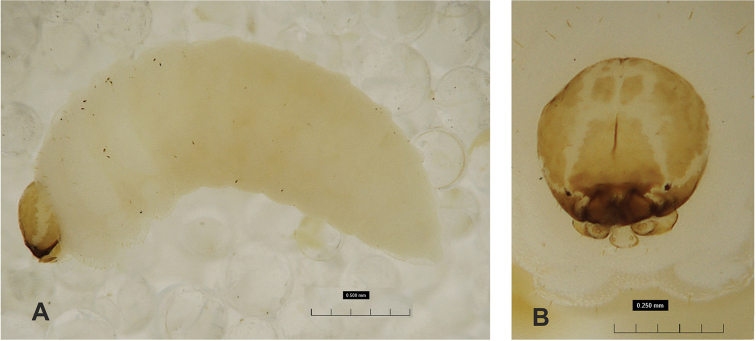
*Gymnetrontibiellum* Desbrochers des Loges mature larva **A** habitus **B** head, dorsal view. Scale bars: 0.5 mm (**A**); 0.25 mm (**B**).

**Figure 2.**
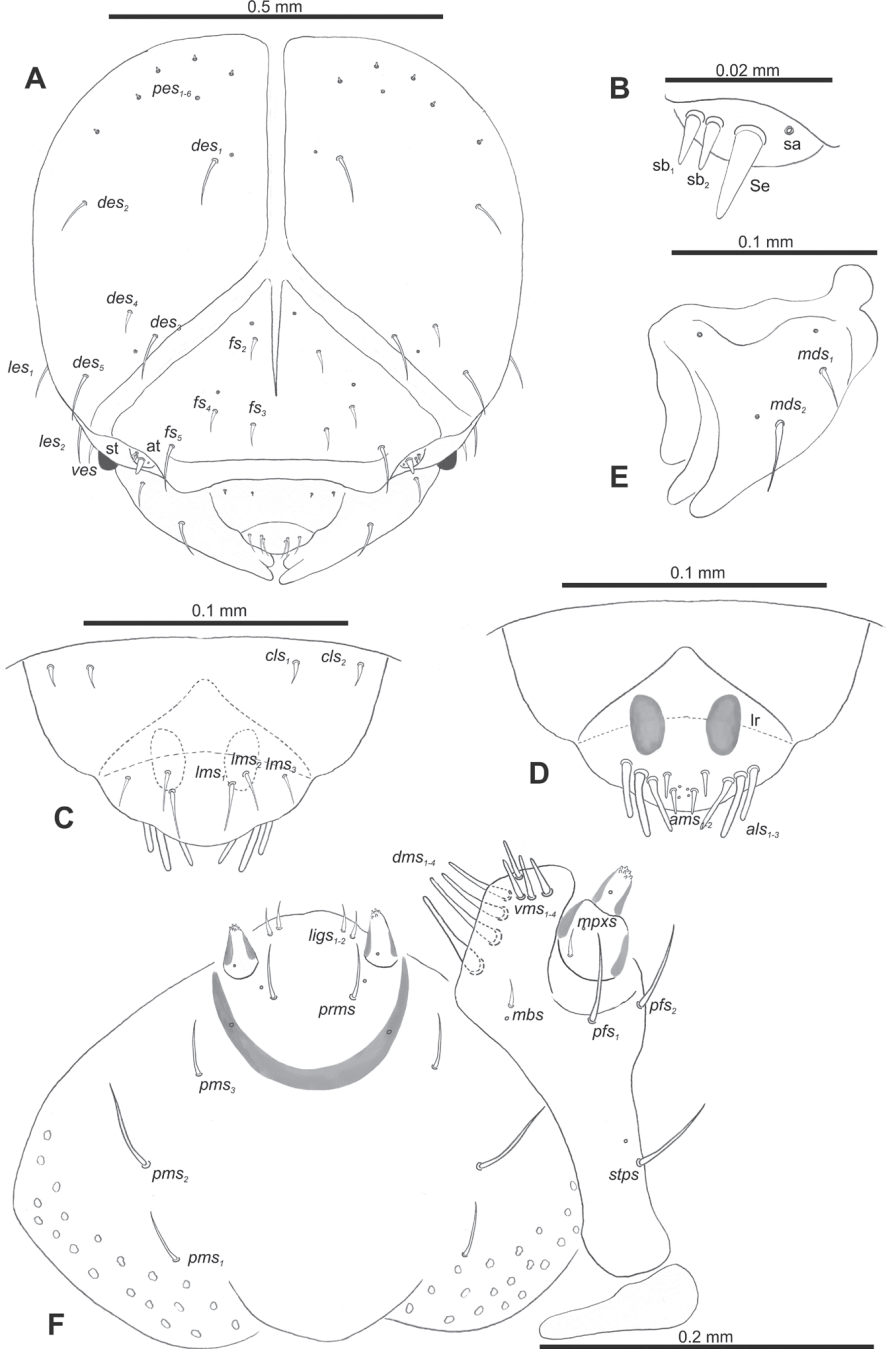
*Gymnetrontibiellum* Desbrochers des Loges mature larva, head and mouth parts **A** head **B** antenna **C** clypeus and labrum **D** epipharynx **E** left mandible **F** maxillolabial complex. Abbreviations: at – antenna, lr – labral rods, sa – sensillum ampullaceum, sb – sensillum basiconicum, Se – sensorium, st – stemma; setae: *als* – anteriolateral, *ams* – anteromedial, *cls* – clypeal, *des* – dorsal epicranial, *dms* – dorsal malar, *fs* – frontal epicranial, *les* – lateral epicranial, *ligs* – ligular, *lms* – labral, *mbs* – basioventral, *mds* – mandibular dorsal, *mpxs* – maxillary palps, *pes* – postepicranial, *pfs* – palpiferal, *pms* – postmental, *prms* – premental, *stps* – stipital, *ves* – ventral, *vms* – ventral malar.

**Figure 3.**
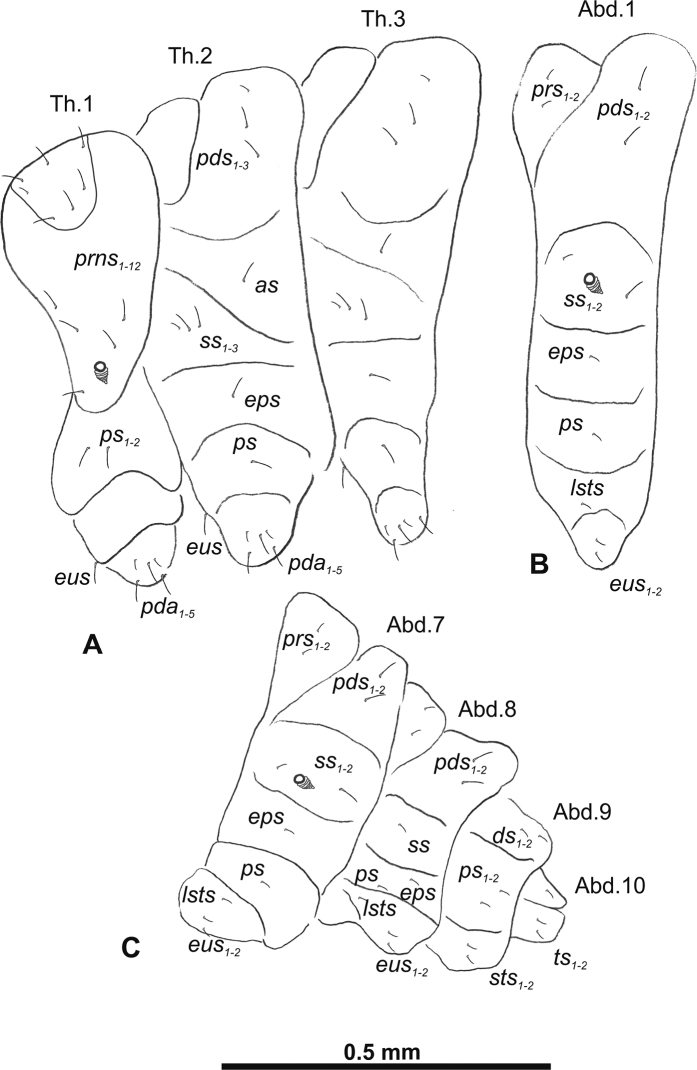
*Gymnetrontibiellum* Desbrochers des Loges mature larva, habitus **A** lateral view of thoracic segments **B** lateral view of abdominal segment I **C** lateral view of abdominal segments VI–X. Abbreviations: Th1–3 – numbers of thoracic segments, Ab1–10 – numbers of abdominal segments, setae: *as* – alar, *ds* – dorsal, *eps* – epipleural, *eus* – eusternal, *lsts* – laterosternal, *pda* – pedal, *pds* – postdorsal, *prns* – pronotal, *prs* – prodorsal, *ss* – spiracular, *ps* – pleural, *sts* – sternal, *ts* – terminal.

(Figs 1A, B, 2A–F, 3A–C). ***Measurements*** (in mm). Body length: 2.25–2.50. The widest point in the body (meso- and metathorax) measures up to 0.95. Head width: 0.42–0.45.

***General*.** Body elongate, slender, weakly curved, rounded in cross section (Fig. 1A).

***Colouration*.** Head pale brown (Fig. 1B). All thoracic and abdominal segments white, cuticle smooth (Fig. 1A).

***Vestiture*.** Setae on body thin, yellowish, distinctly different in length (minute to very short or long).

***Head capsule*** (Figs 1B, 2A). Head suboval, endocarinal line present, extending for 2/3 of length of frons. Frontal sutures on head very broad and distinct. Stemma, in the form of a very small pigmented spot with convex cornea. *Des_1_* long, located in middle of central part of epicranium; *des_2_* medium; *des_3_* long, located anteriorly on epicranium close to border with frontal suture; *des_4_* short; *des_5_* long, located anterolaterally above stemma (Fig. 2A). *Fs_1_* absent; *fs_2_* short, located medially; *fs_3_* short; *fs_4_* short, located anteriorly; and *fs_5_* long, located anterolaterally, close to antenna (Fig. 2A). *Les_1_* and *les_2_* as long as *des_5_*; one short *ves*. Epicranial area with six postepicranial setae.

***Antennae*** membranous and distinctly convex basal membranous article bearing one relatively long conical sensorium and three sensilla of different types: two basiconical and one ampullaceum (Fig. 2B).

***Clypeus*** (Fig. 2C) ~ 3× as wide as long with two medium *cls*, located posterolaterally, without sensillum; fused to labrum.

***Mouth parts*.** Labrum (Fig. 2C) ~ 2× as wide as long, with three piliform *lms*, relatively long, of almost equal length; *lms_1_* located anteromedially, *lms_2_* located partly close to clypeus, and *lms_3_* located anterolaterally. Epipharynx (Fig. 2D) with three very long digitate *als*, almost identical in length; with two piliform, medium *ams*; without *mes*; labral rods indistinct, irregular in shape. Mandibles (Fig. 2E) with two relatively long, piliform *mds*, located in distinct holes. Maxilla (Fig. 2F): stipes with one *stps*, two *pfs* and with one *mbs* and one sensillum, *stps* and both *pfs_1–2_* relatively long; mala with four elongate, digitate *dms*; four *vms*, almost equal in length. Maxillary palpi with two palpomeres; length ratio of basal and distal palpomeres: 1:0.6. Praelabium (Fig. 2F) oval, with one long *prms*; ligula with sinuate margin and two short *ligs*; premental sclerite broad, well visible. Postlabium (Fig. 2F) with three *pms*, medium *pms_1_* located medially, elongated *pms_2_* located laterally, and medium *pms_3_* located anterolaterally; membranous area sparsely and finely asperate.

***Thorax*.** Prothorax (Fig. 3A) with 11 long and one short to minute *prns*, small pigmented dorsal sclerite present with five long and one short *prns*, this sclerite subdivided into two triangular plates medially; two long *ps*; and two short to very short *eus*. Mesothorax (Fig. 3A) without *prs*, two long and one short *pds*; one long *as*; two long and one very short to minute *ss*; one long *eps*; one long *ps*; and two short *eus*. Chaetotaxy of metathorax (Fig. 3A) almost identical to that of mesothorax. Each pedal area of thoracic segments well separated, with three long and two short *pda*.

***Abdomen*.** Spiracles on abdominal segments I–VI close to anterior margin, functional, spiracles on abdominal segment VII not functional. Abdominal segments I–VII (Fig. 3B, C) with two minute *prs*; two long *pds*; one long and one very short to minute *ss*; one short *eps*; one short *ps*; one short *lsts*; and two very short and sometimes one additional minute *eus*. Abdominal segment VIII (Fig. 3C) with two minute *prs*; two long *pds*; one very short to minute *ss*; one short *eps*; one short *ps*; one short *lsts*; and two very short and sometimes one additional minute *eus*. Abdominal segment IX (Fig. 3C) with two short *ds*; two short *ps*; and two very short *sts*. Abdominal segment X (Fig. 3C) with two minute setae (*ts*).

##### Description of pupa

**Figure 4.**
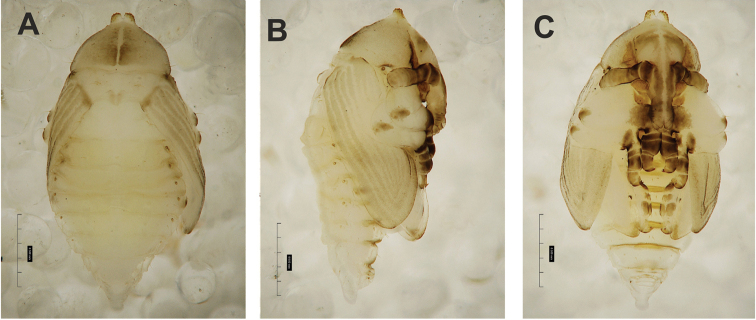
*Gymnetrontibiellum* Desbrochers des Loges pupa habitus **A** ventral view **B** lateral view **C** dorsal view. Scale bars: 0.5 mm.

**Figure 5.**
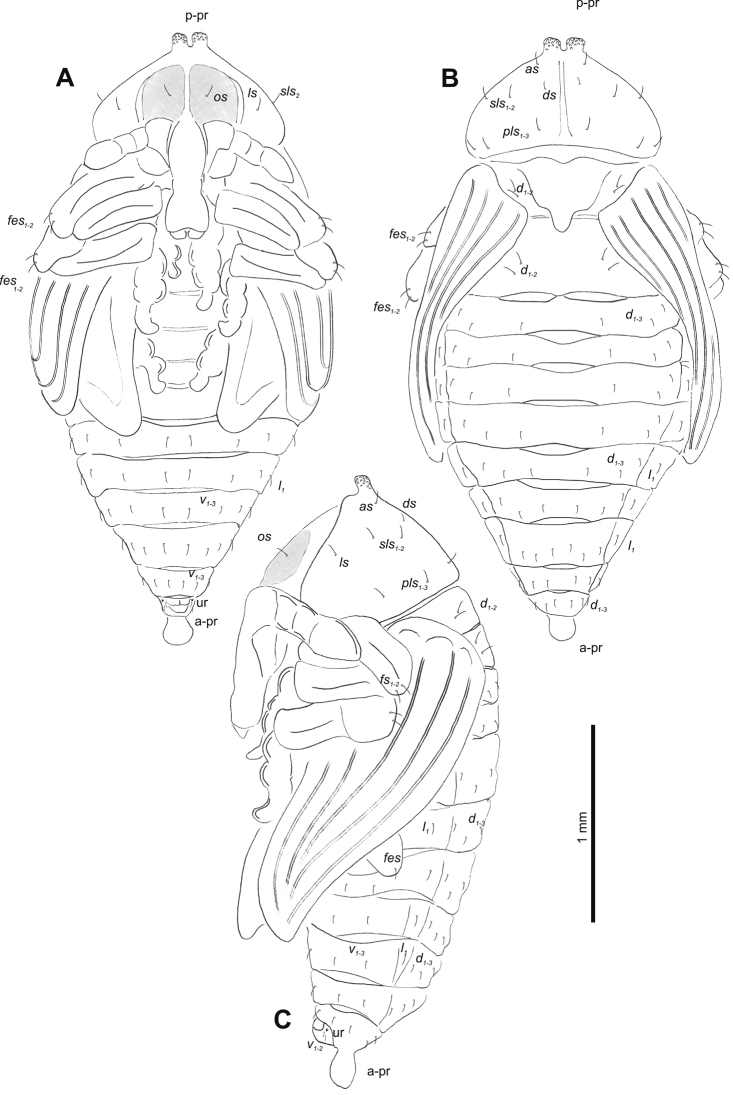
*Gymnetrontibiellum* Desbrochers des Loges pupa habitus **A** ventral view **B** dorsal view **C** lateral view. Abbreviations: a–pr – abdominal protuberances, p–pr – pronotal protuberances, ur – urogomphi; setae: *as* – apical, *d* – dorsal, *ds* – discal, *fes* – femoral, *l*, *ls* – lateral, *os* – orbital, *pls* – posterolateral, *sls* – superlateral, *v* – ventral.

(Figs 4A–C, 5A–C). ***Measurements*** (in mm). Body length: 1.87–2.50. Body width: 0.62–1.25. Thorax width: 0.67–1.12.

***Body*.** Brownish, pronotal protuberances (p-pr) sclerotized, covered with conical asperities; apical parts of femora, head, rostrum and pronotum darker than rest of body. Rostrum moderately slender. Pronotal protuberances fused at base. Pronotum 1.5× as wide as long. Mesonotum slightly smaller than metanotum. Urogomphi in form of minute sclerotized protuberances, almost invisible. Abdominal segment VIII dorsally with rounded, prominent abdominal protuberance (a-pr) (Fig. 5A–C).

***Chaetotaxy*.** Sparse, setae short to medium, transparent. Head with one medium *os*. Rostrum without setae (Fig. 5B). Pronotum with one *as*, one *ds*, two *sls*, one *ls* and three *pls* almost equal in length. Dorsal parts of meso- and metathorax with two setae of various length, placed medially. Apex of pro- and mesofemora with two medium-sized *fes*, metafemora with one seta (Fig. 5A–C). Abdominal segments I–VIII with three short setae of equal length dorsally: first placed medially, the next two more laterally. Each lateral part of abdominal segments I–VIII with single, medium-sized seta. Ventral parts of abdominal segments I–VIII with three medium-sized setae. Abdominal segment IX with two minute setae ventrally (Fig. 5A–C).

##### Biological notes.

The immature stages of *G.tibiellum* were collected from capsules of *Veronicaanagallis-aquatica* L. Previously, nothing was known about the biology of this species. The adults are active from mid-April following the appearance of the host plants. Oviposition takes place from early June until mid-August. The presence of larvae inside the seed capsules is readily detected from the dark colour of the deposited frass. The biologies of *G.tibiellum* and *G.veronicae* are very similar but no competition between these two weevil species has been observed in over 500 dissected seeds capsules where they occur in syntopy.

##### Remarks and comparative notes.

*Gymnetrontibiellum* is widely distributed in the south-eastern part of central Europe, Italy, the Balkans, Caucasus, Anatolia and the Middle East (Alonso-Zarazaga et al. 2017). The adults of this species are very closely related to *G.veronicae*, from which they differ by the shape of the rostra and the penis (Caldara 2008a). This close relationship was confirmed here by several characters which the immature stages have in common, although differences in several other characters of both larvae and pupae readily discriminate these two species.

#### ﻿ Gymnetron veronicae

Taxon classificationAnimaliaColeopteraCurculionidae

(Germar, 1821)

A26141BA-034F-5AA3-AB8C-24BAFDD7D4E9

##### Material examined.

Serbia, Gornji Milanovac, Donja Vrbava, GPS 44°1.663'N, 20°34.496'E, 370 m, ex *Veronicaanagallis-aquatica*, 20.06.2020, leg. Toševski (4 larvae and 4 pupae).

##### Description of mature larva

**Figure 6.**
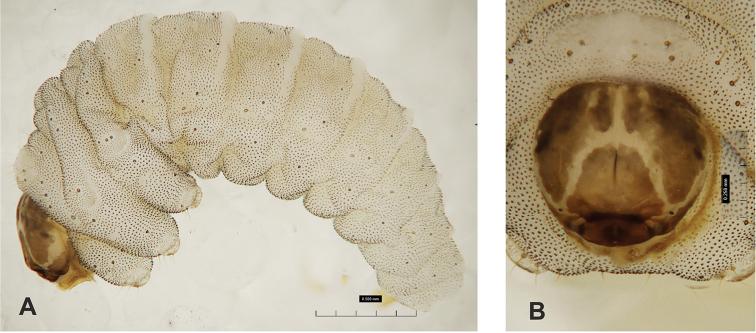
*Gymnetronveronicae* (Germar) mature larva **A** habitus **B** head, dorsal view. Scale bars: 0.5 mm (**A**); 0.25 mm (**B**).

**Figure 7.**
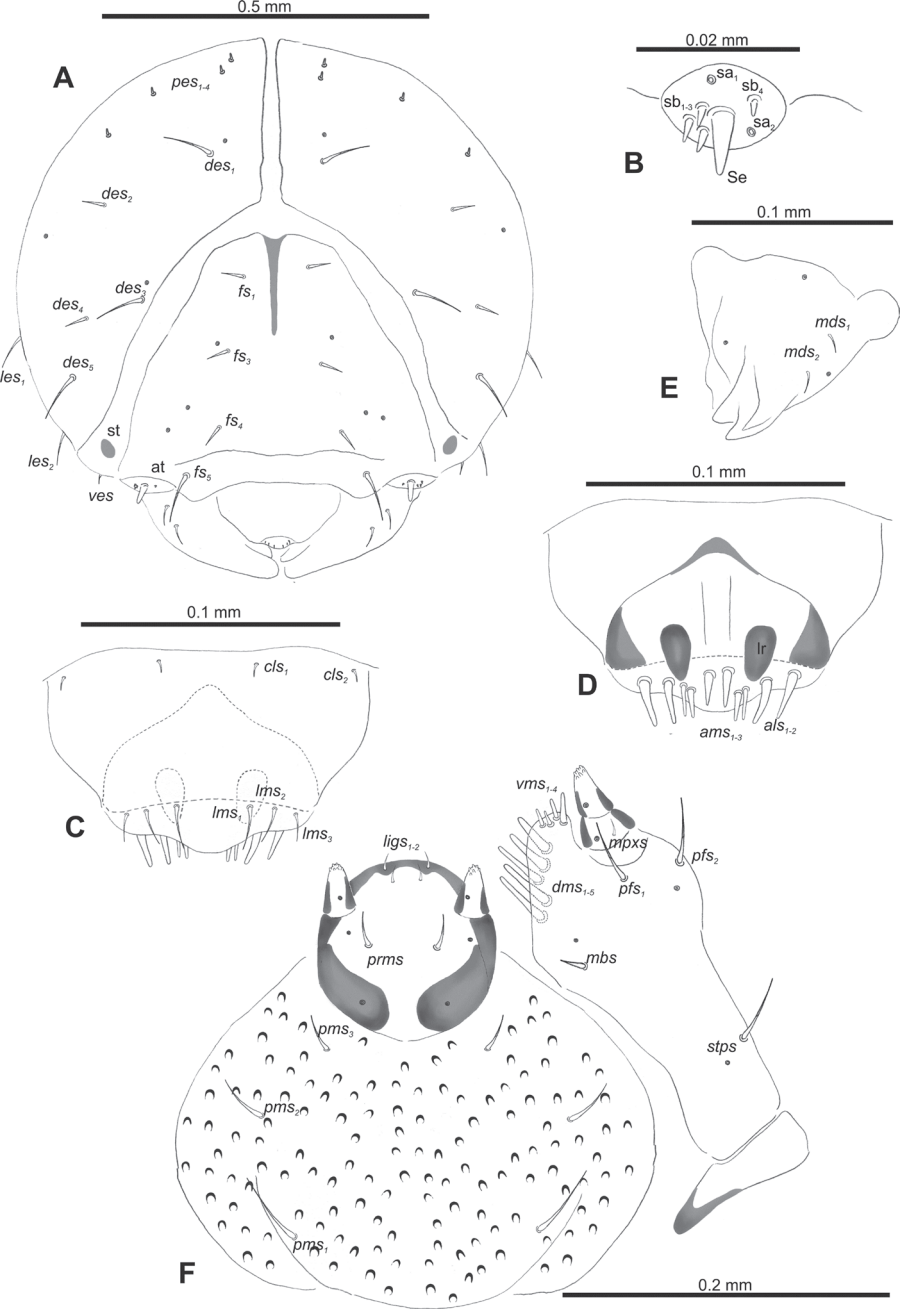
*Gymnetronveronicae* (Germar) mature larva, head and mouth parts **A** head **B** antenna **C** clypeus and labrum **D** epipharynx **E** left mandible **F** maxillolabial complex. Abbreviations: at – antenna, lr – labral rods, sa – sensillum ampullaceum, sb – sensillum basiconicum, Se – sensorium, st – stemma; setae: *als* – anteriolateral, *ams* – anteromedial, *cls* – clypeal, *des* – dorsal epicranial, *dms* – dorsal malar, *fs* – frontal epicranial, *les* – lateral epicranial, *ligs* – ligular, *lms* – labral, *mbs* – basioventral, *mds* – mandibular dorsal, *mpxs* – maxillary palps, *pes* – postepicranial, *pfs* – palpiferal, *pms* – postmental, *prms* – premental, *stps* – stipital, *ves* – ventral, *vms* – ventral malar.

**Figure 8.**
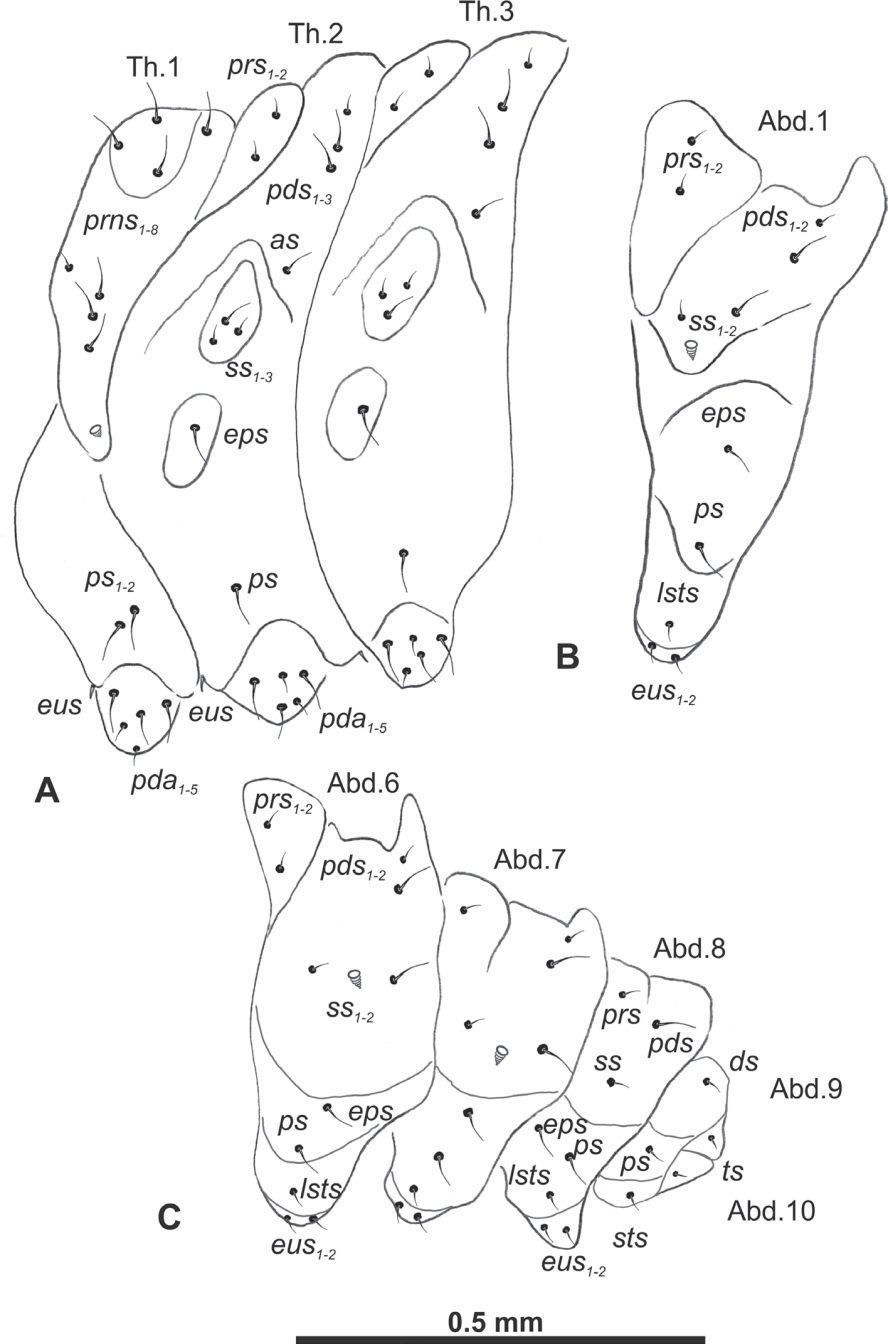
*Gymnetronveronicae* (Germar) mature larva, habitus **A** lateral view of thoracic segments **B** lateral view of abdominal segment I **C** lateral view of abdominal segments VI–X. Abbreviations: Th1–3 – numbers of thoracic segments, Ab1–10 – numbers of abdominal segments; setae: *as* – alar, *ds* – dorsal, *eps* – epipleural, *eus* – eusternal, *lsts* – laterosternal, *pda* – pedal, *pds* – postdorsal, *prns* – pronotal, *prs* – prodorsal, *ss* – spiracular, *ps* – pleural, *sts* – sternal, *ts* – terminal.

(Figs 6A, B, 7A–F, 8A–C). ***Measurements*** (in mm). Body length: 2.16–3.00. The widest point in the body (meso- and metathorax) measures up to 1.00. Head width: 0.50–0.53.

***General*.** Body elongate, slender, weakly curved, rounded in cross section (Fig. 6A).

***Colouration*.** Head dark brown (Fig. 6B). All thoracic and abdominal segments white with numerous reddish or brown asperities (Fig. 6A).

***Vestiture*.** Setae on body thin, orange, distinctly different in length (minute to very short or long).

***Head capsule*** (Figs 6B, 7A). Head suboval, flattened laterally, endocarinal line present, clearly extending to half the length of frons. Frontal sutures on head very broad and distinct. Stemma, in the form of a very small pigmented spot with convex cornea. *Des_1_* long, located in middle of the central part of epicranium; *des_2_* short, placed medially; *des_3_* relatively long, located anteriorly on epicranium close to border with frontal suture; *des_4_* short, placed above frontal suture; *des_5_* long, located anterolaterally (Fig. 7A). *Fs_1_* short; *fs_2_* absent; *fs_3_* located medially; *fs_4_* short, located anteriorly; and *fs_5_* long, located anterolaterally, close to antenna (Fig. 7A). *Les_1_* and *les_2_* as long as *des_5_*; one *ves* minute. Epicranial area with four postepicranial setae.

***Antennae*** membranous and distinctly convex basal membranous article bearing one relatively long conical sensorium and six sensilla different in length (four basiconica and two ampullacea) (Fig. 7B).

***Clypeus*** (Fig. 7C) ~ 3× as wide as long with two medium *cls*, located posterolaterally, without sensillum; fused to labrum.

***Mouth parts*.** Labrum (Fig. 7C) ~ 4× as wide as long, three piliform *lms*, relatively long but of different lengths; *lms_1_* located anteromedially, *lms_2_* located partly close to clypeus, and *lms_3_* located anterolaterally, *lms_1_* and *lms_2_* relatively elongate, *lms_3_* short. Epipharynx (Fig. 7D) with two very long digitate *als*, almost identical in length; with three *ams* of different length, *ams_1_* and *ams_2_* piliform and short, *ams_3_* digitate and enlarged in middle; without *mes*; labral rods indistinct, irregular in shape. Mandibles (Fig. 7E) with two relatively long, piliform *mds*, located in distinct holes. Maxilla (Fig. 7F): stipes with one *stps*, two *pfs* and one minute *mbs* and one sensillum, *stps* and both *pfs* relatively long; mala with five medium, digitate *dms*; four *vms*, of different lengths, two setae very short, and two setae minute. Maxillary palpi with two palpomeres; length ratio of basal and distal palpomeres: 1:0.5. Praelabium (Fig. 7F) suboval-shaped, with one medium *prms*; ligula with sinuate margin and two very short *ligs*; premental sclerite broad, well visible. Postlabium (Fig. 7F) with three *pms: pms_1_* very long; *pms_2_* short, located medially; *pms_3_* located laterally; membranous area densely and finely asperate.

***Thorax*.** Prothorax (Fig. 8A) with seven long and one short *prns*, small pigmented dorsal sclerite present with three long *prns*, this sclerite subdivided into two triangular plates medially; two long *ps*; and two short to very short *eus*. Mesothorax (Fig. 8A) with two very short to minute *prs*; one short and two long *pds*; one long *as*; one long and two very short to minute *ss*; one long *eps*; one long *ps*; and two short *eus*. Chaetotaxy of metathorax (Fig. 8A) almost identical to that of mesothorax. Each pedal area of thoracic segments well separated, with three long and two very short to minute *pda*.

***Abdomen*.** Spiracles on abdominal segments I–VI close to the anterior margin and functional, spiracles on abdominal segment VII not functional, and abdominal segment VIII with atrophied spiracles. Abdominal segments I–VII (Fig. 8B, C) with two minute *prs* (segment VII with one *prs*); one long and one minute *pds*; one long and one very short to minute *ss*; one long *eps*; one relatively long *ps*; one short *lsts*; and two very short and sometimes one additional minute *eus*. Abdominal segment VIII (Fig. 8C) with one minute *prs*; one long *pds*; one very short to minute *ss*; one long *eps*; one relatively long *ps*; one short *lsts*; and two very short and sometimes one additional minute *eus*. Abdominal segment IX (Fig. 8C) with one relatively long *ds*; one relatively long *ps*; and one short to very short *sts*. Abdominal segment X (Fig. 8C) with one very short seta (*ts*).

##### Description of pupa

**Figure 9.**
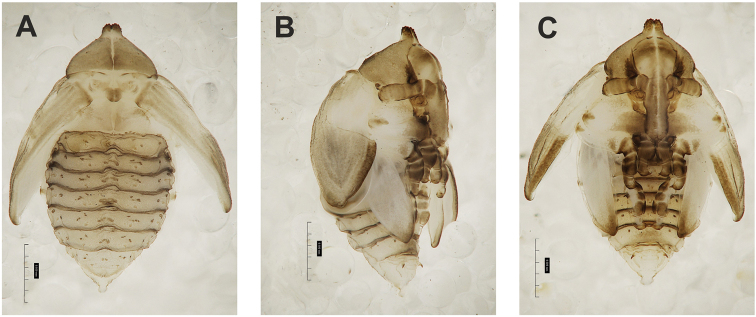
*Gymnetronveronicae* (Germar) pupa habitus **A** ventral view **B** lateral view **C** dorsal view. Scale bars: 0.5 mm.

**Figure 10.**
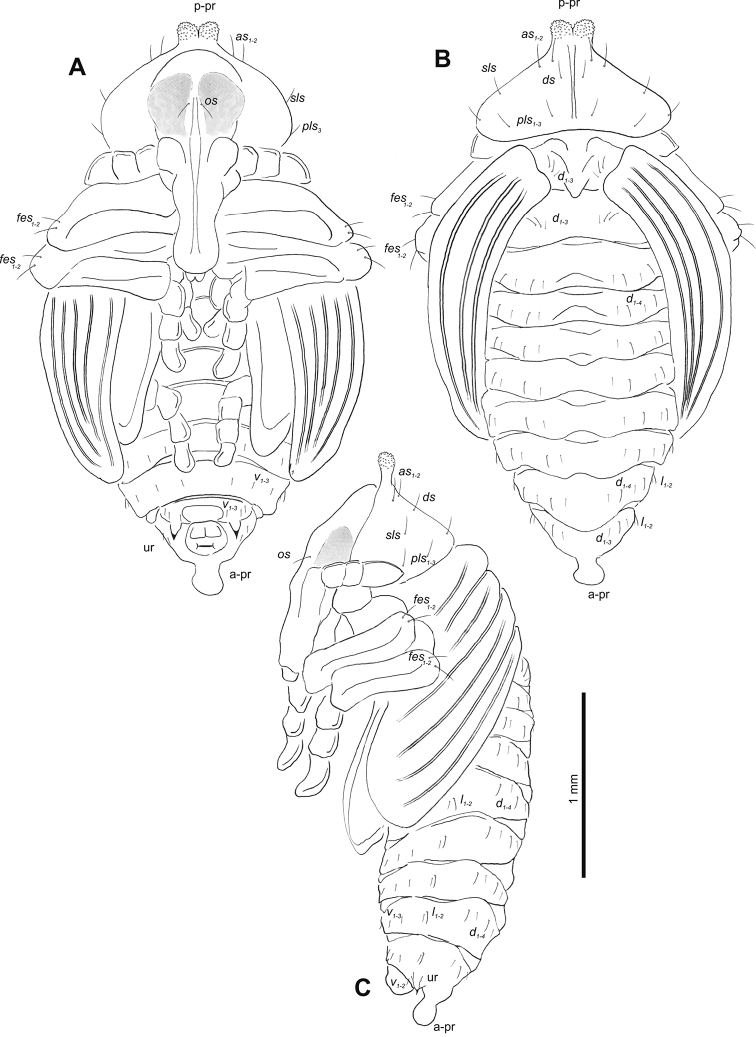
*Gymnetronveronicae* (Germar) pupa habitus **A** ventral view **B** dorsal view **C** lateral view. Abbreviations: a–pr – abdominal protuberances, p–pr – pronotal protuberances, ur – urogomphi; setae: *as* – apical, *d* – dorsal, *ds* – discal, *fes* – femoral, *l* – lateral, *os* – orbital, *pls* – posterolateral, *sls* – superlateral, *v* – ventral.

(Figs 9A–C, 10A–C). ***Measurements*** (in mm). Body length: 2.12–2.32. Body width: 1.25–1.23, Thorax width: 0.70–0.80.

***Body*.** Brownish, pronotal protuberances (p-pr) sclerotized, covered with conical asperities; apical parts of femora, head, rostrum and pronotum darker than rest of body. Rostrum moderately slender. Pronotal protuberances almost completely fused. Pronotum twice as wide as long. Mesonotum slightly smaller than metanotum. Urogomphi short, conical, with sclerotized apices. Abdominal segment VIII with rounded, prominent abdominal protuberance dorsally (a-pr) (Fig. 10A–C).

***Chaetotaxy*.** Sparse, setae short to medium, transparent. Head with one medium *os*. Rostrum without setae (Fig. 10B). Pronotum with two elongate *as*, one *ds*, one *sls*, and three *pls*, all of almost equal length. Dorsal parts of meso- and metathorax with three setae of various length, situated medially. Apex of femora with two medium-sized *fes* (Fig. 10A–C). Abdominal segments I–VIII with four medium to short setae placed in horizontal line medially. Each lateral part of abdominal segments I–VIII with two setae of various size. Ventral parts of abdominal segments I–VIII with three medium setae. Abdominal segment IX with two minute setae ventrally (Fig. 10A–C).

##### Biological notes.

The larva was already known to feed on the ovary of *Veronicabeccabunga* L, where it pupates and develops to the adult stage, and the adult was also collected on *V.anagallis-aquatica* L. and *V.scutellata* L. (Hoffmann 1958; Koch 1992; Sprick 1997). We can now confirm that at least *V.anagallis-aquatica* L. must be another host plant. The biology of this weevil species is the same as that of *G.tibiellum*.

##### Remarks and comparative notes.

The adult of this species, widely distributed throughout Europe (Alonso-Zarazaga et al. 2017), is closely related to *G.tibiellum*, but with which it is sympatric only in south-eastern Europe. The two species differ mainly in the shapes of the rostra and the penis. Examination of the larvae confirms the relationship between them: they share the praedorsal segment on the abdominal segments with two *pds*, the epicranium with *fs_3_* and the labral setae in one line. However, the larva of *G.veronicae* differs from that of *G.tibiellum* by the cuticle of the body covered with numerous reddish or brown asperities and setae emerging from black spots, the dark brown not pale yellow head, and the epipharynx with two (not three) *als* and three (not two) *ams*. The pupae also have many characters in common (see the key), but clearly differ by the number of setae *as*, *ls* and *sls* on the pronotum, those on the meso- and metathorax, and on the dorsal parts of abdominal segments I–VII.

#### ﻿ Gymnetron rotundicolle

Taxon classificationAnimaliaColeopteraCurculionidae

Gyllenhal, 1838

0B85A3D0-BEBE-5695-9CD8-3C3E2C410886

##### Material examined.

Serbia, Kalna, GPS 43°24.673'N, 22°25.737'E, 365 m, ex *Veronicahederophylla*, 20.06.2020, leg. Toševski (20 larvae); Serbia, Zemun, GPS 44°51.313'N, 20°22.625'E, 105 m, ex *V.opaca*, 19.06.2020, leg. Toševski (4 larvae and 2 pupae).

##### Description of mature larva

**Figure 11.**
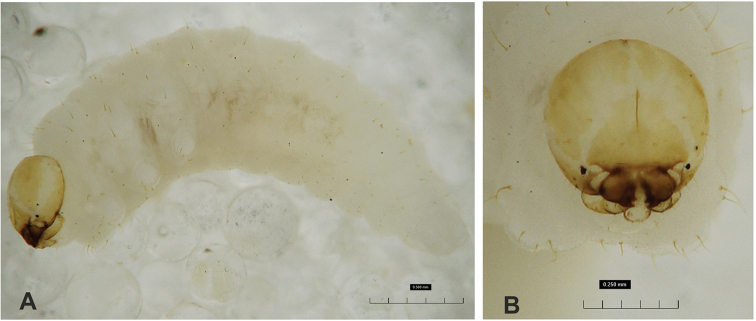
*Gymnetronrotundicolle* Gyllenhal mature larva **A** habitus **B** head, dorsal view. Scale bars: 0.5 mm (**A**); 0.25 mm (**B**).

**Figure 12.**
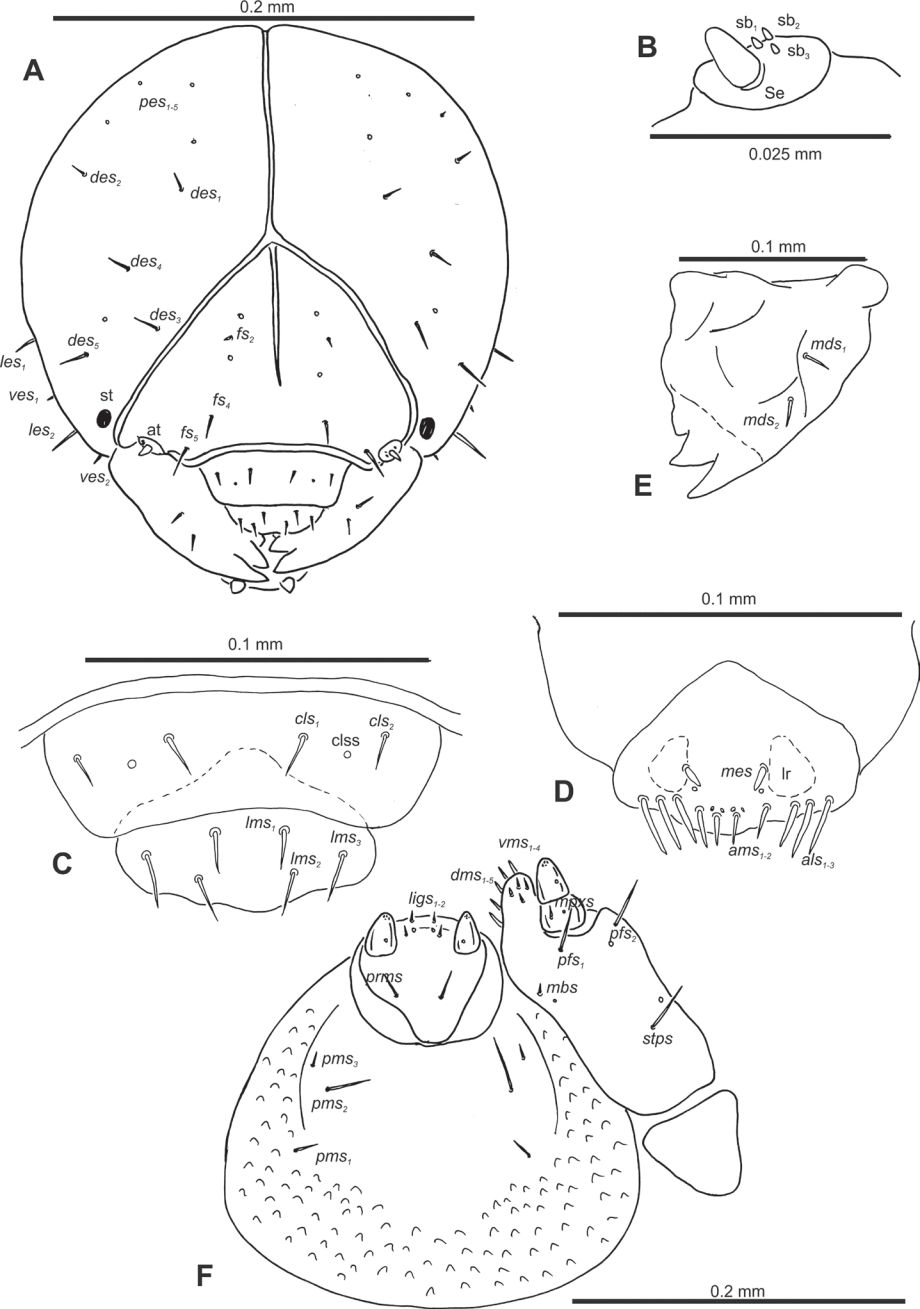
*Gymnetronrotundicolle* Gyllenhal mature larva, head and mouth parts **A** head **B** antenna **C** clypeus and labrum **D** epipharynx **E** left mandible **F** maxillolabial complex. Abbreviations: at – antenna, clss – clypeal sensillium, lr – labral rods, sb – sensillum basiconicum, Se – sensorium, st – stemma; setae: *als* – anteriolateral, *ams* – anteromedial, *cls* – clypeal, *des* – dorsal epicranial, *dms* – dorsal malar, *fs* – frontal epicranial, *les* – lateral epicranial, *ligs* – ligular, *lms* – labral, *mbs* – basioventral, *mds* – mandibular dorsal, *mes* – median, *mpxs* – maxillary palps, *pes* – postepicranial, *pfs* – palpiferal, *pms* – postmental, *prms* – premental, *stps* – stipital, *ves* – ventral, *vms* – ventral malar.

**Figure 13.**
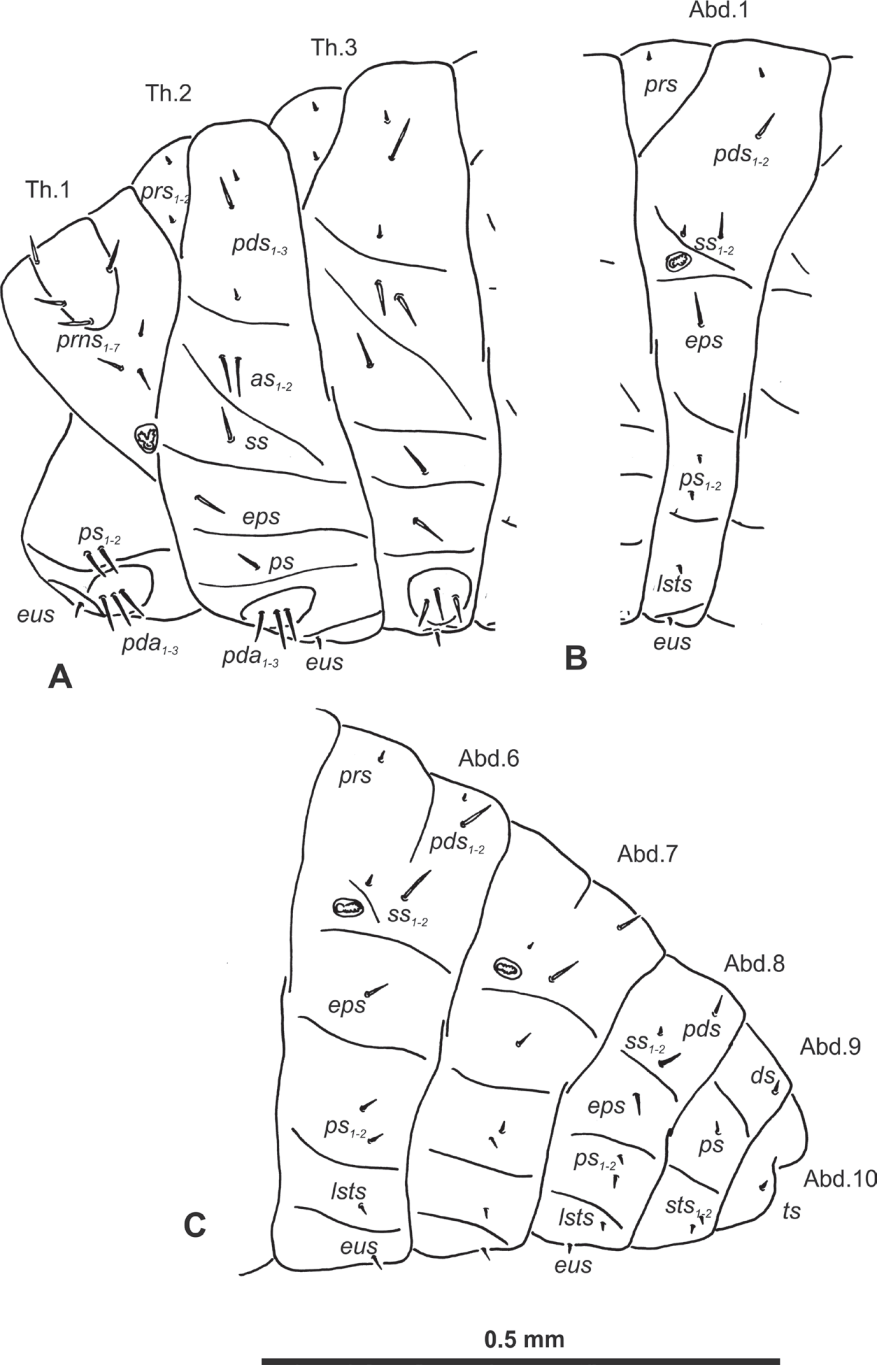
*Gymnetronrotundicolle* Gyllenhal mature larva, habitus **A** lateral view of thoracic segments **B** lateral view of abdominal segment I **C** lateral view of abdominal segments VI–X. Abbreviations: Th1–3 – numbers of thoracic segments, Ab1–10 – numbers of abdominal segments; setae: *as* – alar, *ds* – dorsal, *eps* – epipleural, *eus* – eusternal, *lsts* – laterosternal, *pda* – pedal, *pds* – postdorsal, *prns* – pronotal, *prs* – prodorsal, *ss* – spiracular, *ps* – pleural, *sts* – sternal, *ts* – terminal.

(Figs 11A, B, 12A–F, 13A, B). ***Measurements*** (in mm). Body length: 2.20–2.33. The widest point in the body (meso- and metathorax) measures up to 0.86. Head width: 0.36–0.50.

***General*.** Body relatively elongate, distinctly curved, rounded in cross section (Fig. 11A).

***Colouration*.** Head pale yellow (Fig. 11B). All thoracic and abdominal segments white, cuticle smooth (Fig. 11A).

***Vestiture*.** Setae on body thin, transparent, distinctly different in length (minute to very short or medium).

***Head capsule*** (Figs 11B, 12A). Head suboval, endocarinal line present, shorter than half the length of frons. Frontal sutures on head of medium width, distinct. Stemma, in form of distinct, black pigmented spot with convex cornea. *Des_1_* short, located in middle of central part of epicranium; *des_2_* short, located in middle of central part of epicranium; medium size *des_3_* located anteriorly on epicranium close to border with frontal suture; *des_4_* short, located between *des_2_* and *des_3_*; *des_5_* of medium size, located anterolaterally (Fig. 12A). *Fs_1_* absent; *fs_2_* very short to minute, located medially; *fs_3_* absent; *fs_4_* medium, located anteriorly; and *fs_5_* relatively long, located anterolaterally, close to antenna (Fig. 12A). *Les_1_* and *les_2_* as long as *des_5_*; two *ves* short. Epicranial area with four postepicranial setae (*pes*).

***Antennae*** membranous and distinctly convex basal membranous article bearing one relatively long conical sensorium and three sensilla basiconica (Fig. 12B).

***Clypeus*** (Fig. 12C) ~ 3× as wide as long with two relatively long *cls: cls_1_* located posterolaterally, *cls_2_* located posteromedially, and one sensillum between setae; not fused with labrum.

***Mouth parts*.** Labrum (Fig. 12C) ~ 3× as wide as long, with three piliform *lms*, relatively long, of almost equal length; *lms_1_* located posteromedially, close to clypeus, *lms_2_* located anteromedially, and *lms_3_* located anterolaterally. Epipharynx (Fig. 12D) with three very long digitate *als*, almost identical in length, two piliform *ams* almost equal in length and one *mes*; labral rods indistinct, enlarged anteriorly. Mandibles (Fig. 12E) with two relatively long, piliform *mds*, located in distinct holes. Maxilla (Fig. 12F): stipes with one *stps*, two *pfs*, one *mbs* and sensillum, *stps* and *pfs_1–2_* long, *mbs* very short; mala with five relatively long, digitate *dms*; four *vms*, different in length, one setae very short, and three setae minute. Maxillary palpi with two palpomeres; length ratio of basal and distal palpomeres: 1:0.5. Praelabium (Fig. 12F) oval, with one relatively long *prms*; ligula with sinuate margin and two very short *ligs* and one sensillum; premental sclerite broad, readily visible at sides but almost invisible in middle. Postlabium (Fig. 12F) with three *pms*, very long *pms_2_*, and very short to short *pms_1_* and *pms_3_*, all located laterally; membranous area sparsely and finely asperate.

***Thorax*.** Prothorax (Fig. 13A) with six relatively long and one short to very short *prns*, pigmented dorsal sclerite present with four relatively long *prns*, this sclerite subdivided into two triangular plates medially; two relatively long *ps*; and one short *eus*. Mesothorax (Fig. 13A) with two very short to minute *prs*, one relatively long and two short to very short *pds*; one relatively long *as*; two relatively long *ss*; one relatively long *eps*; one relatively long *ps*; and one short *eus*. Chaetotaxy of metathorax (Fig. 13A) almost identical to that of mesothorax. Each pedal area of thoracic segments well separated, with three long *pda*.

***Abdomen*.** Spiracles on abdominal segments I–VI close to the anterior margin and functional, spiracles on abdominal segment VII not functional, and abdominal segment VIII with atrophied spiracles. Abdominal segments I–VI (Fig. 13B, C) with one very short to minute *prs*; one relatively long and one very short *pds*; one relatively long and one very short to minute *ss*; one relatively long *eps*; two very short *ps*; one very short *lsts*; and one very short to minute *eus*. Abdominal segments VII–VIII (Fig. 13C) without *prs*; with one relatively long *pds*; one very short to minute *ss*; one relatively long *eps*; two very short *ps*; one very short *lsts*; and one very short to minute *eus*. Abdominal segment IX (Fig. 13C) with one very short *ds*; one very short *ps*; and two very short *sts*. Abdominal segment X (Fig. 13C) with one very short to minute seta (*ts*).

##### Description of pupa

**Figure 14.**
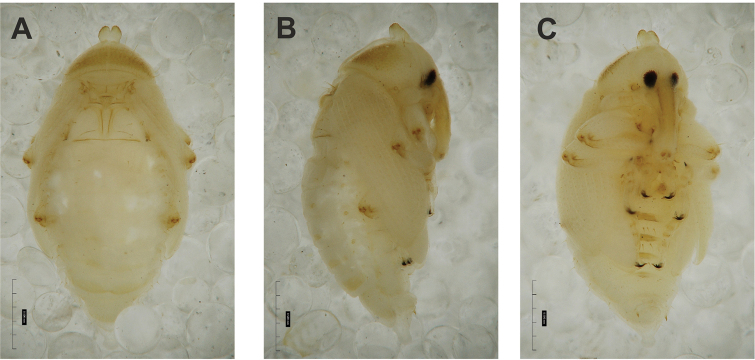
*Gymnetronrotundicolle* Gyllenhal pupa habitus **A** ventral view **B** lateral view **C** dorsal view. Scale bars: 0.5 mm.

**Figure 15.**
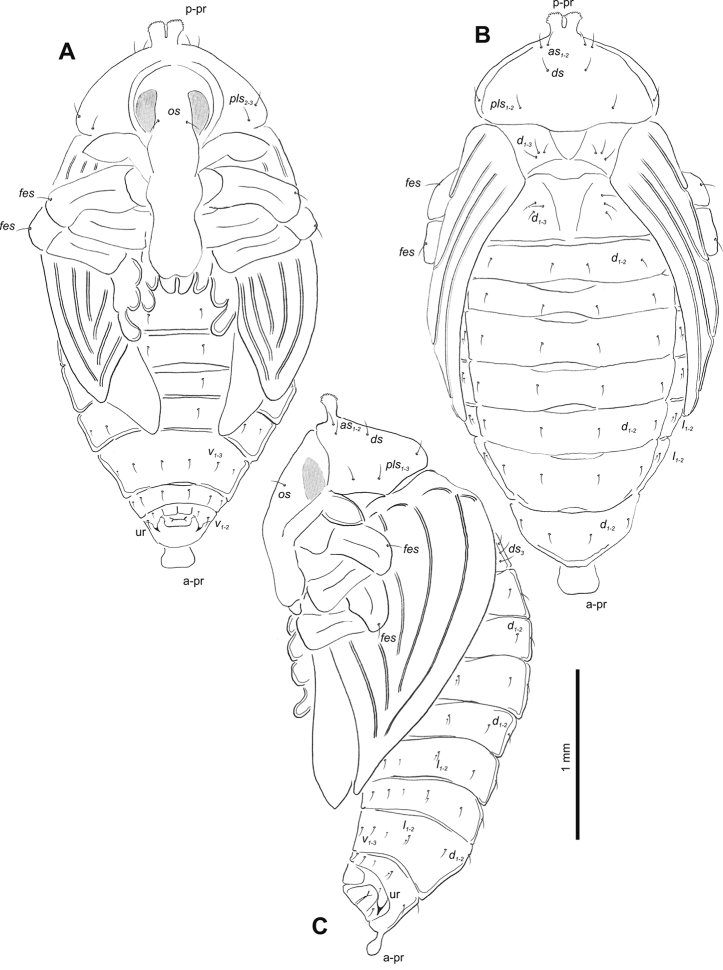
*Gymnetronrotundicolle* Gyllenhal pupa habitus **A** ventral view **B** dorsal view **C** lateral view. Abbreviations: a–pr – abdominal protuberances, p–pr – pronotal protuberances, ur – urogomphi; setae: *as* – apical, *d* – dorsal, *ds* – discal, *fes* – femoral, *l*, *ls* – lateral, *os* – orbital, *pls* – posterolateral, *v* – ventral.

(Figs 14A–C, 15A–C). ***Measurements*** (in mm). Body length: 2.20–2.37. Body width: 1.12–1.42, Thorax width: 0.70–0.85.

***Body*.** Yellowish, pronotal protuberances (p-pr) weakly sclerotized, with serrated margins; apical parts of femora brownish. Rostrum slender. Pronotum twice as wide as long. Pronotal protuberances fused at basis. Mesonotum slightly smaller than metanotum. Urogomphi reduced, conical, with sclerotized apex. Abdominal segment VIII with rounded, prominent abdominal protuberance dorsally (a-pr) (Fig. 15A–C).

***Chaetotaxy*.** Sparse, setae short to medium, transparent. Head with one short *os*. Rostrum without setae (Fig. 14B). Pronotum with two *as*, one *ds*, and three *pls* equal in length. Dorsal parts of meso- and metathorax with three setae of different length, situated medially. Apex of femora with one medium *fes* (Fig. 15A–C). Abdominal segments I–VIII with two short, equally long setae dorsally: one situated medially, the other mediolaterally. All dorsal abdominal setae almost equal in length, short. Each lateral part of abdominal segments I–VIII with two setae of various length (one short, one minute). Ventral parts of abdominal segments I–VIII with three medium setae. Abdominal segment IX with two very short setae ventrally (Fig. 15A–C).

##### Biological notes.

The adults of *G.rotundicolle* were previously recorded as collected on two species of *Veronica: V. persica* Poiret in Italy and Switzerland (Caldara 2008b; Germann et al. 2013), and *V.chamaedrys* L. in the Czech Republic and Slovakia (Krátký and Trnka 2012; Krátký 2013). The reports of *Veronicahederifolia* L. and *V.opaca* Fr. as host plants of this weevil are new data. The adults appear in early spring (mid-March), feeding on the upper leaves of newly growing shoots of the host. Oviposition takes place in the seed capsules, in which the larvae complete their development. The presence of larvae inside seed capsules can be detected from the dark colour of their frass.

##### Remarks and comparative notes.

The first findings of this originally central Asian species in many countries of central and southern Europe (Italy, Switzerland, France, Germany, Czech Republic, Slovakia, Hungary, Poland) have been reported in many faunistic papers during the last 15 years (Strejček 2007; Caldara 2008b; Krátký and Trnka 2012; Krátký 2013; Germann et al. 2013; Reibnitz 2013; Podlussány et al. 2017; Wanat and Ruta 2018; Nolte and Haag 2019). These papers indicate with a high degree of certainty that this species only recently colonized areas where a few years ago it was absent, in contrast to its host plants (Caldara 2008b; Germann et al. 2013).

#### ﻿ Gymnetron melanarium

Taxon classificationAnimaliaColeopteraCurculionidae

(Germar, 1821)

39922FEF-B664-5F58-971A-13E99C4031CF

##### Material examined.

Serbia, Pirot, Ponor, GPS 43°11.013'N, 22°25.067'E, 686 m, ex Veronicaaustriacasubsp.jacquinii, 20.06.2020, leg. Toševski (35 larvae and 11 pupae).

##### Description of mature larva

**Figure 16.**
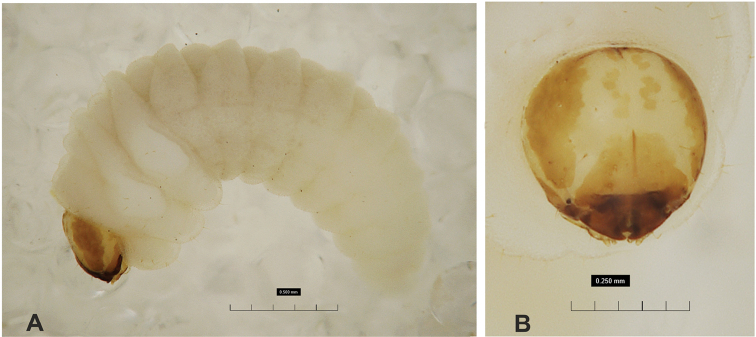
*Gymnetronmelanarium* (Germar) mature larva **A** habitus **B** head, dorsal view. Scale bars: 0.5 mm (**A**); 0.25 mm (**B**).

**Figure 17.**
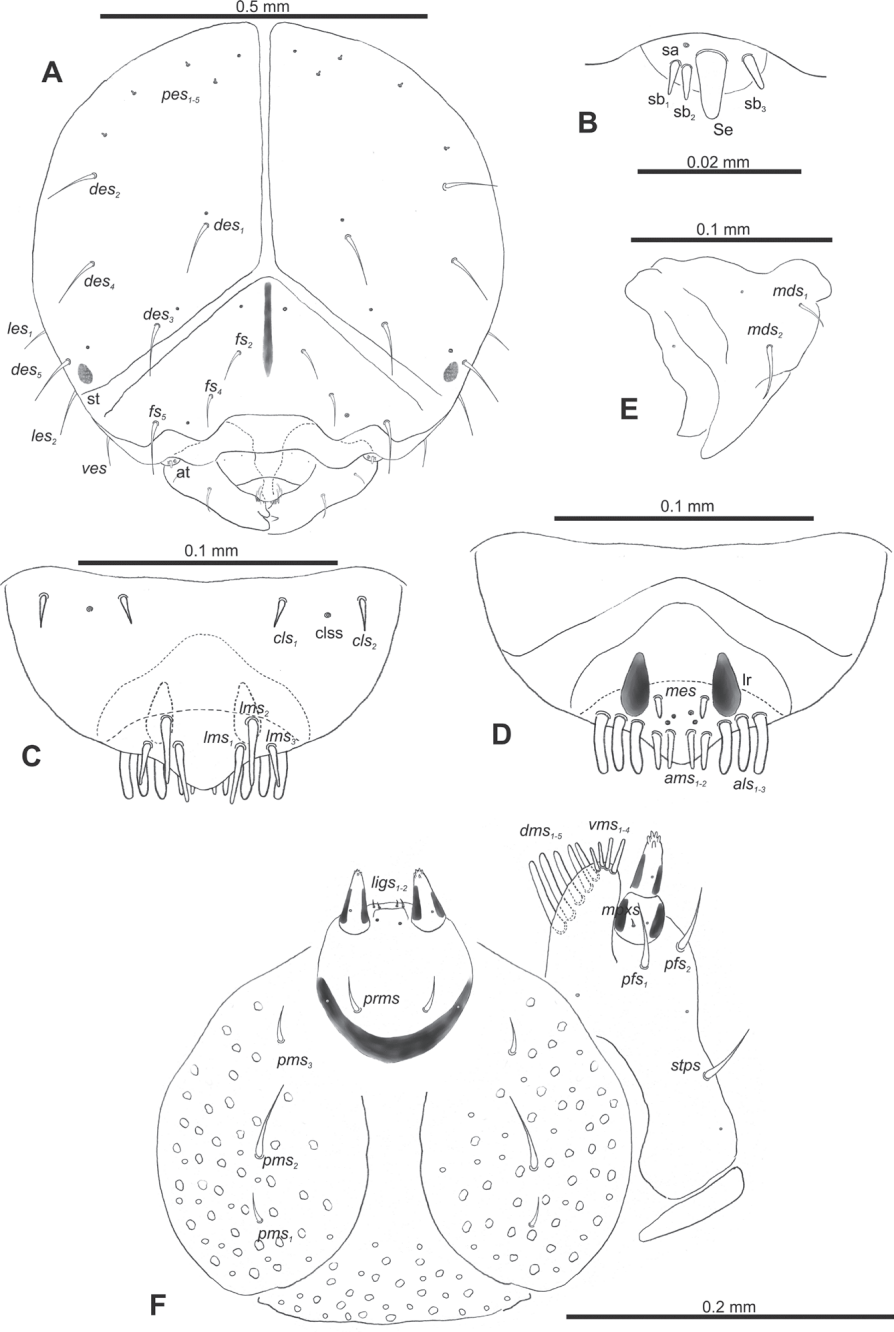
*Gymnetronmelanarium* (Germar) mature larva, head and mouth parts **A** head **B** antenna **C** clypeus and labrum **D** epipharynx **E** left mandible **F** maxillolabial complex. Abbreviations: at – antenna, clss – clypeal sensillium, lr – labral rods, sa – sensillum ampullaceum, sb – sensillum basiconicum, Se – sensorium, st – stemma; setae: *als* – anteriolateral, *ams* – anteromedial, *cls* – clypeal, *des* – dorsal epicranial, *dms* – dorsal malar, *fs* – frontal epicranial, *les* – lateral epicranial, *ligs* – ligular, *lms* – labral, *mds* – mandibular dorsal, *mes* – median, *mpxs* – maxillary palps, *pes* – postepicranial, *pfs* – palpiferal, *pms* – postmental, *prms* – premental, *stps* – stipital, *ves* – ventral, *vms* – ventral malar.

**Figure 18.**
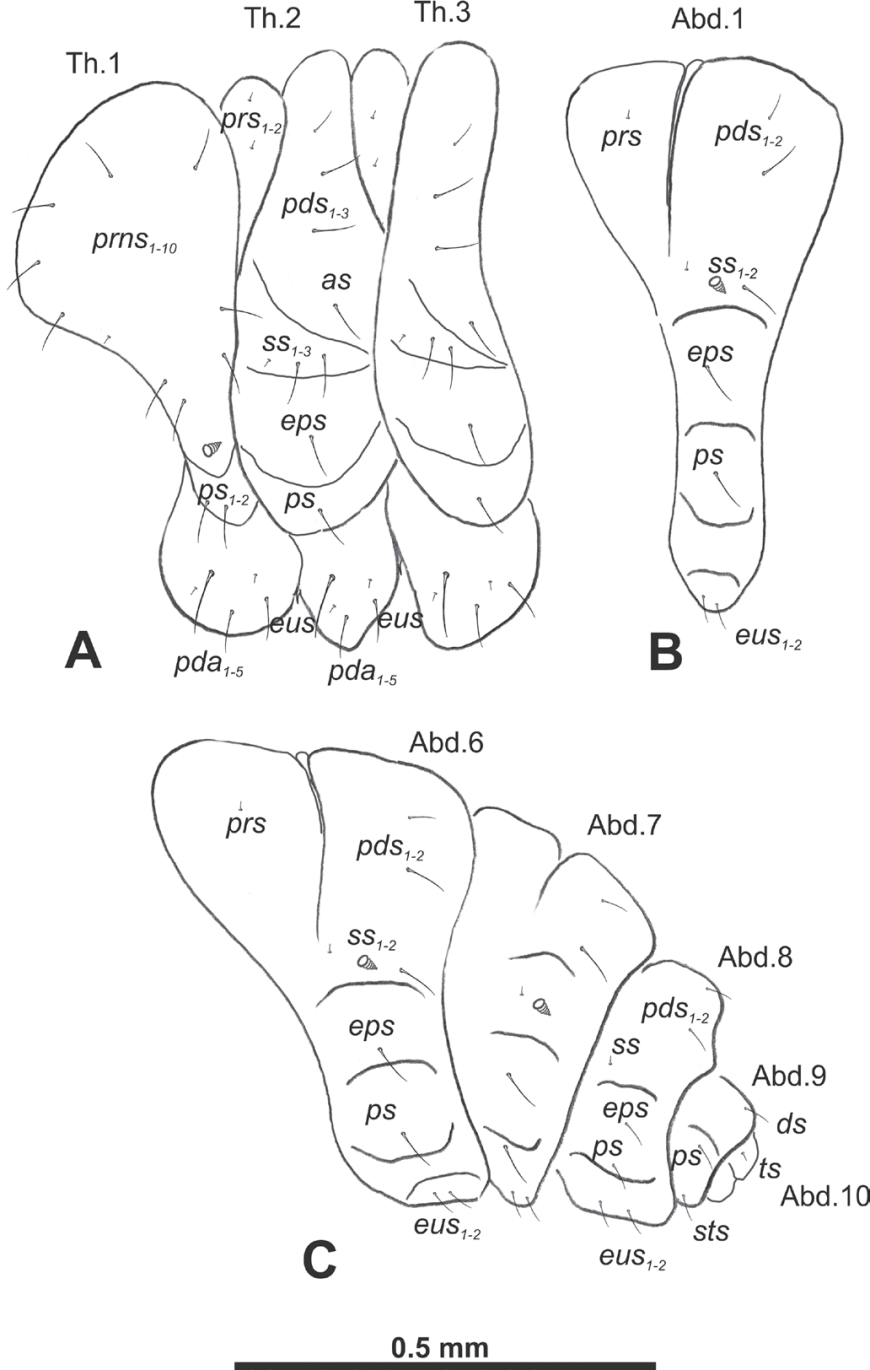
*Gymnetronmelanarium* (Germar) mature larva, habitus **A** lateral view of thoracic segments **B** lateral view of abdominal segment I **C** lateral view of abdominal segments VI–X. Abbreviations: Th1–3 – numbers of thoracic segments, Ab1–10 – numbers of abdominal segments; setae: *as* – alar, *ds* – dorsal, *eps* – epipleural, *eus* – eusternal, *lsts* – laterosternal, *pda* – pedal, *pds* – postdorsal, *prns* – pronotal, *prs* – prodorsal, *ss* – spiracular, *ps* – pleural, *sts* – sternal, *ts* – terminal.

(Figs 16A, B, 17A–F, 18A, B). ***Measurements*** (in mm). Body length: 2.33–2.66. The widest point in the body (meso- and metathorax) measures up to 1.00. Head width: 0.50–0.53.

***General*.** Body elongate, slender, weakly curved, rounded in cross section (Fig. 16A).

***Colouration*.** Head pale yellow (Fig. 16B). All thoracic and abdominal segments smooth (Fig. 16A).

***Vestiture*.** Setae on body thin, yellow, distinctly different in length (minute to very short or long).

***Head capsule*** (Figs 16B, 17A). Head almost oval, endocarinal line present, extending distinctly to 3/4 of the length of frons. Frontal sutures on head distinct. Stemma, in form of pigmented spot with convex cornea. *Des_1_* medium, located in middle of central part of epicranium; medium *des_2_*; medium *des_3_* located anteriorly on epicranium, close to border with frontal suture; medium *des_4_*; medium *des_5_* placed laterally (Fig. 17A). *Fs_1_* absent; *fs_2_* medium, located medially; *fs_3_* absent; *fs_4_* medium, located anteriorly; and *fs_5_* long, located anterolaterally, close to antenna (Fig. 17A). *Les_1_* medium and *les_2_* as long as *des_5_*; single *ves* medium. Epicranial area with six postepicranial setae.

***Antennae*** membranous and distinctly convex basal membranous article bearing one relatively long conical sensorium and four sensilla: three basiconica and single ampullaceum (Fig. 17B).

***Clypeus*** (Fig. 17C) ~ 3× as wide as long with two relatively long *cls*, located posterolaterally, with single sensillum; fused to labrum.

***Mouth parts*.** Labrum (Fig. 17C) ~ 3× as wide as long, with three piliform *lms*, relatively long, *lms_3_* slightly shorter than others; *lms_1_* located anteromedially, *lms_2_* located partly close to clypeus, and *lms_3_* located anterolaterally. Epipharynx (Fig. 17D) with three very long digitate *als*, almost identical in length; with two piliform *ams*, equal in length, and one short, digitate *mes*; labral rods indistinct, narrow. Mandibles (Fig. 17E) with two relatively long, piliform *mds*, located in distinct holes. Maxilla (Fig. 17F): stipes with one *stps*, two *pfs* and one sensillum, without *mbs*; *stps* and both *pfs* long; mala with five moderately elongate digitate *dms*; four *vms*, different in length, two setae short, and two setae very short. Maxillary palpi with two palpomeres; length ratio of basal and distal palpomeres: 1:1.2. Praelabium (Fig. 17F) oval, with one medium *prms*; ligula with rounded margin and two very short *ligs*; premental sclerite broad, well visible. Postlabium (Fig. 17F) with three *pms*, short *pms_1_*, very long *pms_2_* and short *pms_3_*, all located laterally; membranous area densely and distinctly asperate.

***Thorax*.** Prothorax (Fig. 18A) with nine long and one minute *prns*; two long *ps*; and two very short *eus*. Mesothorax (Fig. 18A) with two minute *prs*; one medium and two long *pds*; one long *as*; two long and one minute *ss*; one long *eps*; one long *ps*; and one short *eus*. Chaetotaxy of metathorax (Fig. 18A) almost identical to that of mesothorax. Each pedal area of thoracic segments well separated, with one long, two medium and two very short to minute *pda*.

***Abdomen*.** Spiracles on abdominal segments I–VI placed medially and functional, spiracles on abdominal segment VII not functional, and abdominal segment VIII with atrophied spiracles. Abdominal segments I–VI (Fig. 18B, C) with one minute *prs*; one long and one medium *pds*; one long and one very short to minute *ss*; one long *eps*; one relatively long *ps*; without *lsts* and two very short *eus*. Abdominal segments VII–VIII (Fig. 18C) without *prs*; one long and one medium *pds*; one very short to minute *ss*; one long *eps*; one long *ps*; and two very short *eus*. Abdominal segment IX (Fig. 18C) with one relatively long *ds*; one relatively long *ps*; and one medium *sts*. Abdominal segment X (Fig. 18C) with one very short seta (*ts*).

##### Description of pupa

**Figure 19.**
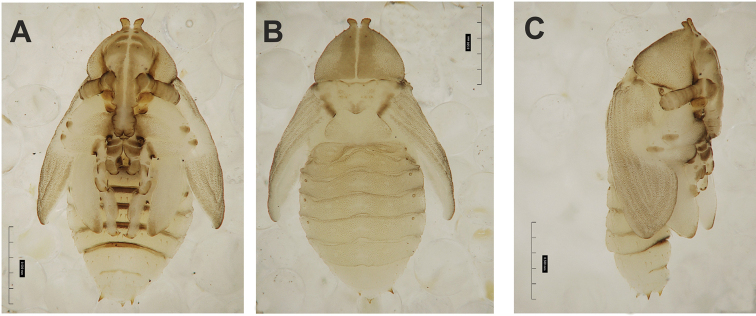
*Gymnetronmelanarium* (Germar) pupa habitus **A** ventral view **B** lateral view **C** dorsal view. Scale bars: 0.5 mm.

**Figure 20.**
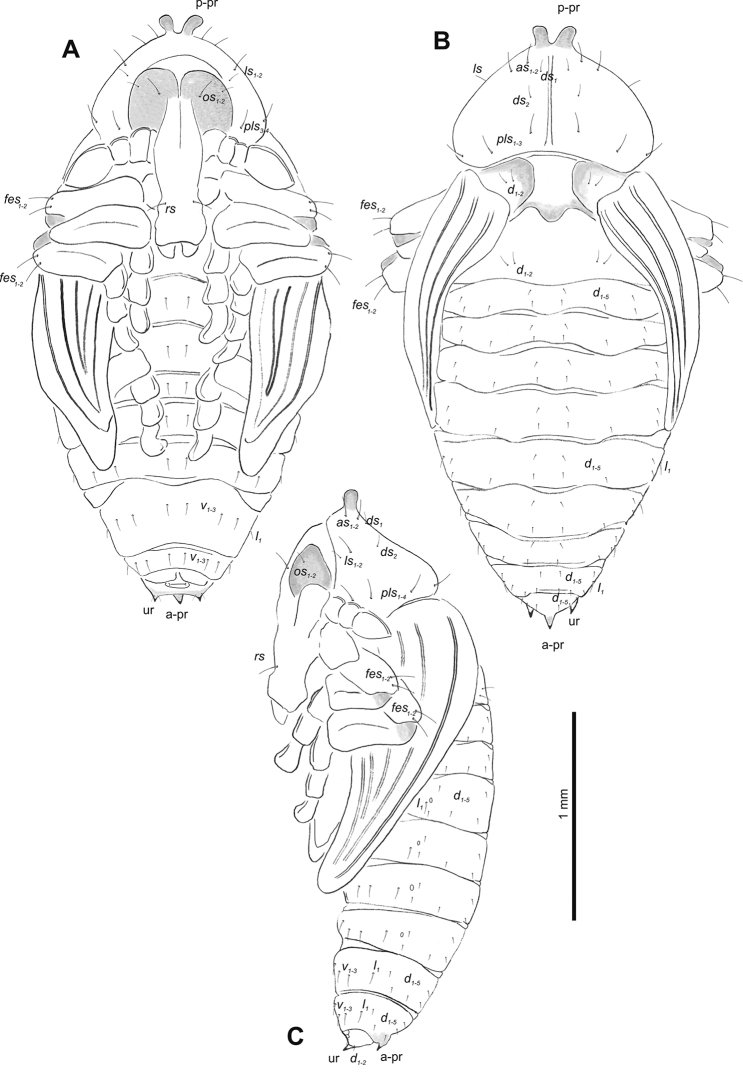
*Gymnetronmelanarium* (Germar) pupa habitus **A** ventral view **B** dorsal view **C** lateral view. Abbreviations: a–pr – abdominal protuberances, p–pr – pronotal protuberances, ur – urogomphi; setae: *as* – apical, *d* – dorsal, *ds* – discal, *fes* – femoral, *l*, *ls* – lateral, *os* – orbital, *pls* – posterolateral, *rs* – rostral, *v* – ventral.

(Figs 19A–C, 20A–C). ***Measurements*** (in mm). Body length: 2.12–2.32. Body width: 1.25–1.32. Thorax width: 0.75–0.82.

***Body*.** Yellowish, pronotal protuberances (p-pr) sclerotized, smooth; head, rostrum, antennae, dorsal parts of meso- and metanotum, and apical parts of femora brownish. Rostrum rather slender. Pronotal protuberances well separated. Pronotum 1.8× as wide as long. Mesonotum slightly smaller than metanotum. Urogomphi reduced, conical, with sclerotized apex. Abdominal segment VIII with conical abdominal protuberance dorsally (a-pr) having acute, sclerotized apex (Fig. 19A–C).

***Chaetotaxy*.** Sparse, setae rather short to moderately elongate, transparent. Head with two *os*, different in length. Rostrum with one *rs*. Setae on head and rostrum straight, as long as those on prothorax (Fig. 20B). Pronotum with two *as*, two *ls*, two *ds* and four *pls*; *ds_1_*_–*2*_ and *ls_2_* slightly shorter than other pronotal setae. Dorsal parts of meso- and metathorax with two setae placed medially. Apex of femora with two *fes* equal in length (Fig. 20A–C). Abdominal segments I–VIII with five short, equally long setae dorsally: first placed antero-medially, the others distributed in regular line along posterior margin of segment. All dorsal abdominal setae short, almost equal in length. Each lateral part of abdominal segments I–VIII with one elongated seta. Ventral parts of abdominal segments I–VIII with three medium setae. Abdominal segment IX with two very short setae ventrally (Fig. 20A–C).

##### Biological notes.

Previously the larva of this species was observed on *Veronicaserpyllifolia* L., on the stems where it produces a small uni- or bilocular gall in which metamorphosis takes place. The adult emerges from the gall at the end of summer and hibernates in the soil (Hustache 1931; Hoffmann 1958). The adult has also been collected on other *Veronica* species such as *V.agrestis* L., V.austriacasubsp.austriaca L., *V.chamaedrys* L., *V.officinalis* L, and *V.teucrium* (L.) D.A. Webb (Hoffmann 1958; Koch 1992; Sprick 1997). In Serbia, the development of *G.melanarium* is restricted to the seed capsules of Veronicaaustriacasubsp.jacquinii (Baumg.) Watzl, which is new information. Nearly 90% of the seed capsules are infested with one or two larvae. The larvae are seed feeders and development occurs in the basal part of the strongly flattened, glossy and glabrous seed capsules with no visible sign of larval presence. Oviposition takes place from mid-May onwards and the new generation of adults emerges during July.

##### Remarks and comparative notes.

This species belongs to a group of very similar species characterized by slender subrectangular elytra, rostrum in lateral view tapered from the antennal insertion to the apex, and short protibiae in the female. There are no particular phylogenetic affinities with the adult (see Caldara 2008a) and pupal stages (abdominal protuberance short, triangular, head with 2 *os*) of the other species described here. By contrast, the larvae share several characters with *G.rotundicolle*, e.g., the praedorsal segment on abdominal segments with one *pds*, the epicranium lacking *fs_3_*, and the conical layout of the labral setae.

#### ﻿ Gymnetron villosulum

Taxon classificationAnimaliaColeopteraCurculionidae

Gyllenhal, 1838

ADB0D1D2-2375-5249-A786-687F3865040A

##### Material examined.

Serbia, Boljetin, GPS 44°30.973'N, 22°0.921'E, 139 m, ex gall *Veronicaanagallis-aquatica*, 16.07.2012, leg. Toševski (3 larvae and 1 pupa).

##### Description of mature larva

**Figure 21.**
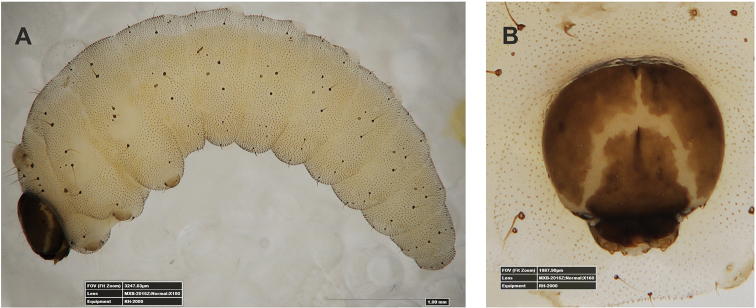
*Gymnetronvillosulum* Gyllenhal mature larva **A** habitus **B** head, dorsal view. Scale bar: 1 mm.

**Figure 22.**
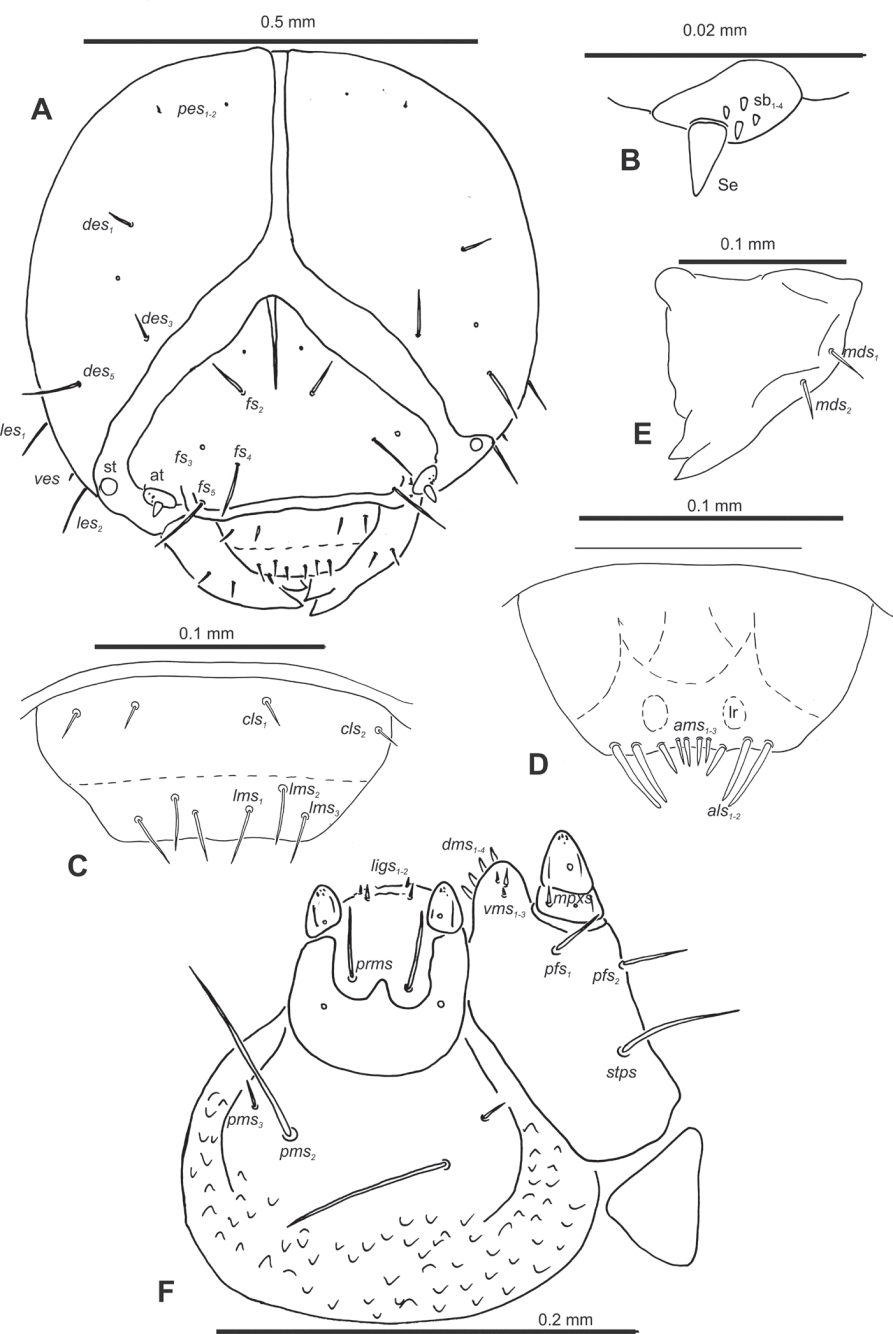
*Gymnetronvillosulum* Gyllenhal mature larva, head and mouth parts **A** head **B** antenna **C** clypeus and labrum **D** epipharynx **E** left mandible **F** maxillolabial complex. Abbreviations: at – antenna, lr – labral rods, sb – sensillum basiconicum, Se – sensorium, st – stemma; setae: *als* – anteriolateral, *ams* – anteromedial, *cls* – clypeal, *des* – dorsal epicranial, *dms* – dorsal malar, *fs* – frontal epicranial, *les* – lateral epicranial, *ligs* – ligular, *lms* – labral, *mds* – mandibular dorsal, *mpxs* – maxillary palps, *pes* – postepicranial, *pfs* – palpiferal, *pms* – postmental, *prms* – premental, *stps* – stipital, *ves* – ventral, *vms* – ventral malar.

**Figure 23.**
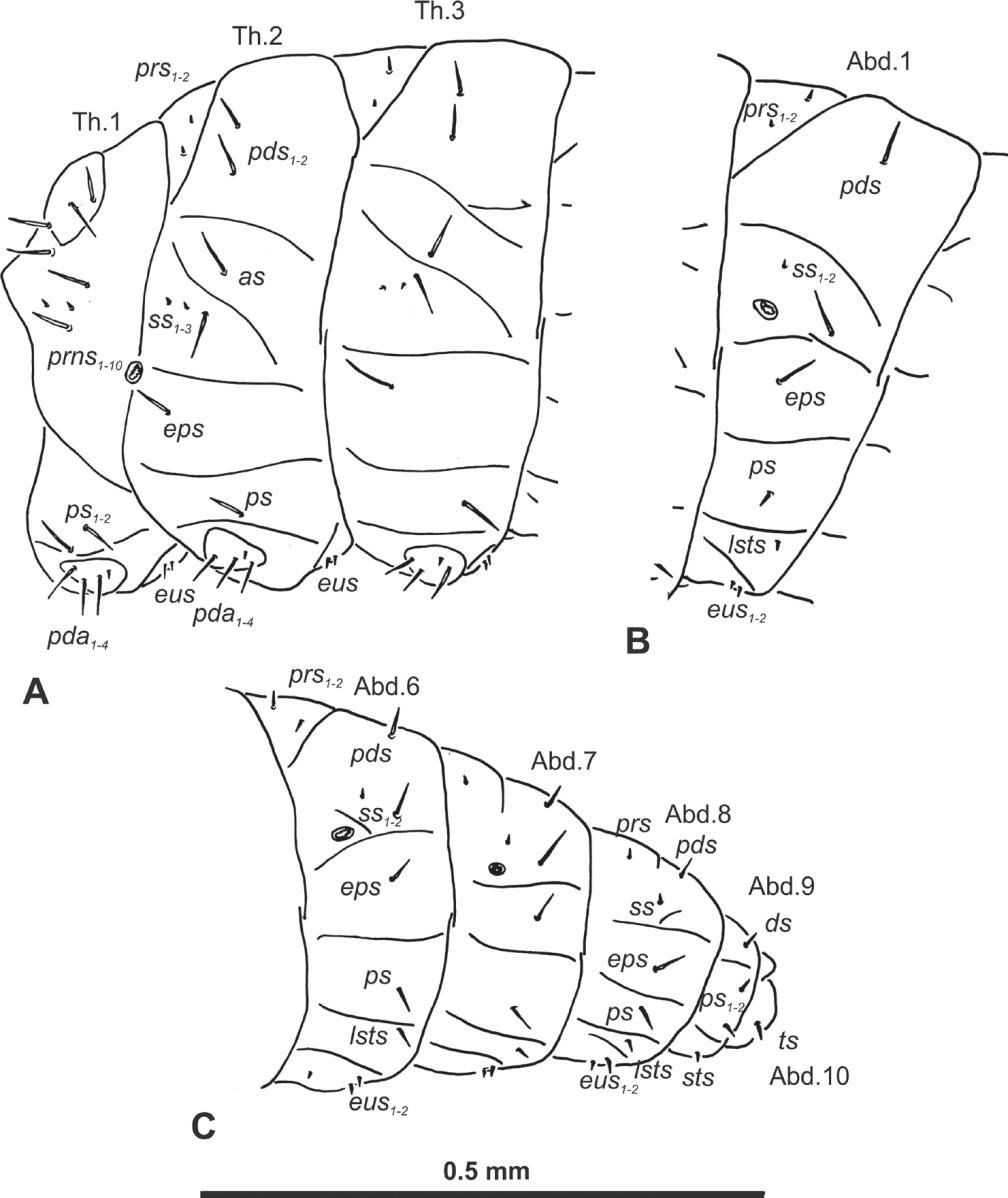
*Gymnetronvillosulum* Gyllenhal mature larva, habitus **A** lateral view of thoracic segments **B** lateral view of abdominal segment I **C** lateral view of abdominal segments VI–X. Abbreviations: Th1–3 – numbers of thoracic segments, Ab1–10 – numbers of abdominal segments; setae: *as* – alar, *ds* – dorsal, *eps* – epipleural, *eus* – eusternal, *lsts* – laterosternal, *pda* – pedal, *pds* – postdorsal, *prns* – pronotal, *prs* – prodorsal, *ss* – spiracular, *ps* – pleural, *sts* – sternal, *ts* – terminal.

(Figs 21A, B, 22A–F, 23A–C). ***Measurements*** (in mm). Body length: 2.25–2.46. The widest point in the body (meso- and metathorax) measures up to 1.20. Head width: 0.40–0.51.

***General*.** Body elongate, slender, weakly curved, rounded in cross section (Fig. 21A).

***Colouration*.** Head dark brown (Fig. 21B). All thoracic and abdominal segments white with many reddish or brown asperities (Fig. 21A).

***Vestiture*.** Setae on body thin, orange, distinctly different in length (minute to very short or long).

***Head capsule*** (Figs 21B, 22A). Head suboval, flattened laterally, endocarinal line present, clearly extending to 1/3 of the length of frons. Frontal sutures on head very broad and distinct. Stemma, in the form of a very small pigmented spot with convex cornea. *Des_1_* short, located in middle of central part of epicranium; *des_2_* absent; relatively long *des_3_* located anteriorly on epicranium close to border with frontal suture; *des_4_* absent; *des_5_* long, located anterolaterally (Fig. 22A). *Fs_1_* absent; *fs_2_* relatively long, located medially; *fs_3_* absent; *fs_4_* relatively long, located anteriorly; and *fs_5_* long, located anterolaterally, close to antenna (Fig. 22A). *Les_1_* and *les_2_* as long as *des_5_*; *ves* short. Epicranial area with two postepicranial setae.

***Antennae*** membranous and distinctly convex basal membranous article bearing one relatively long conical sensorium and four sensilla basiconica (Fig. 22B).

***Clypeus*** (Fig. 22C) ~ 3–4× as wide as long with two relatively long *cls*, located posterolaterally, without sensillum; fused to labrum.

***Mouth parts*.** Labrum (Fig. 22C) ~ 4× as wide as long, with three piliform *lms*, relatively long, almost of equal length; *lms_1_* located anteromedially, *lms_2_* partly located close to clypeus, and *lms_3_* located anterolaterally. Epipharynx (Fig. 22D) with two very long digitate *als*, almost identical in length; with three *ams* of different length, *ams_1_* and *ams_2_* piliform and short, digitate *ams_3_* and enlarged in middle; without *mes*; labral rods indistinct, irregular in shape. Mandibles (Fig. 22E) with two relatively long, piliform *mds*, located in distinct holes. Maxilla (Fig. 22F): stipes with one *stps*, two *pfs* and without *mbs* and sensillum, *stps* and *pfs_1_* long, *pfs_2_* relatively long; mala with four short digitate *dms*; three *vms*, different lengths, one seta very short, and two setae minute. Maxillary palpi with two palpomeres; length ratio of basal and distal palpomeres: 1:0.5. Praelabium (Fig. 22F) oval, with one long *prms*; ligula with sinuate margin and two very short *ligs*; premental sclerite broad, well visible. Postlabium (Fig. 22F) with two *pms*, *pms_1_* absent, short *pms_2_* located laterally and very long *pms_3_* located medially; membranous area sparsely and finely asperate.

***Thorax*.** Prothorax (Fig. 23A) with six long and two very short to minute *prns*, small pigmented dorsal sclerite present with two long *prns*, this sclerite subdivided into two triangular plates medially; two long *ps*; and two short to very short *eus*. Mesothorax (Fig. 23A) with two very short to minute *prs*, two long *pds*; one long *as*; one long and two very short to minute *ss*; one long *eps*; one long *ps*; and two short *eus*. Chaetotaxy of metathorax (Fig. 23A) almost identical to that of mesothorax. Each pedal area of thoracic segments well separated, with three long and one very short to minute *pda*.

***Abdomen*.** Spiracles on abdominal segments I–VI close to the anterior margin and functional, spiracles on abdominal segment VII not functional, and abdominal segment VIII with atrophied spiracles. Abdominal segments I–VI (Fig. 23B, C) with one short and one minute *prs*; one long *pds*; one long and one very short to minute *ss*; one long *eps*; one relatively long *ps*; one short *lsts*; and two very short and sometimes one additional minute *eus*. Abdominal segments VII–VIII (Fig. 23C) with one very short *prs*; one long *pds*; one long and one very short to minute *ss*; one long *eps*; one relatively long *ps*; one short *lsts*; and two very short and sometimes one additional minute *eus*. Abdominal segment IX (Fig. 23C) with one relatively long *ds*; two relatively long *ps*; and one short to very short *sts*. Abdominal segment X (Fig. 23C) with one very short seta (*ts*).

##### Description of pupa

**Figure 24.**
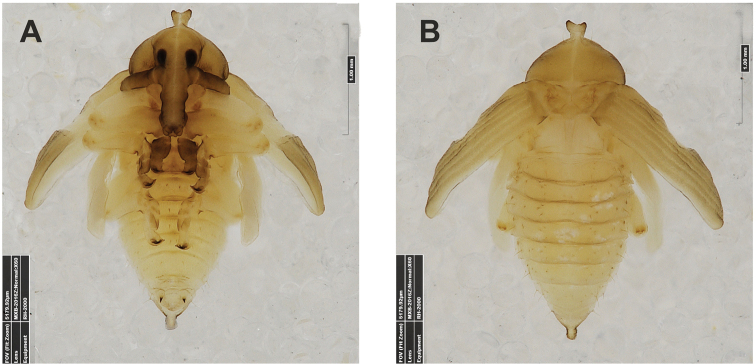
*Gymnetronvillosulum* Gyllenhal pupa habitus **A** ventral view **B** dorsal view. Scale bars: 1 mm.

**Figure 25.**
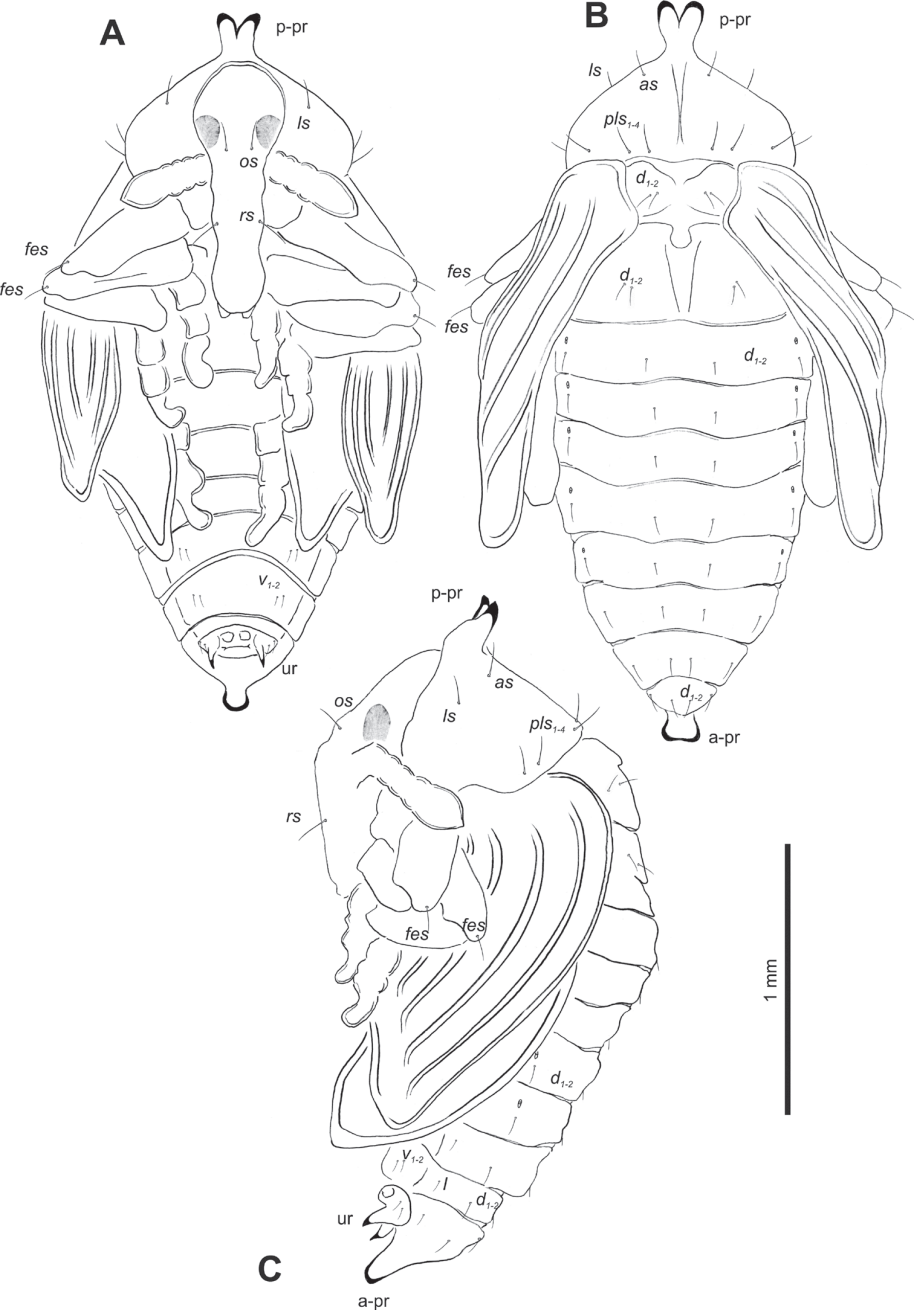
*Gymnetronvillosulum* Gyllenhal pupa habitus **A** ventral view **B** dorsal view **C** lateral view. Abbreviations: a–pr – abdominal protuberances, p–pr – pronotal protuberances, ur – urogomphi; setae: *as* – apical, *d* – dorsal, *ds* – discal, *fes* – femoral, *l*, *ls* – lateral, *os* – orbital, *pls* – posterolateral, *rs* – rostral, *v* – ventral.

(Figs 24A–C, 25A–C). ***Measurements*** (in mm). Body length: 2.24–2.73. Body width: 1.30–1.55. Thorax width: 0.82–0.88.

***Body*.** Brownish, pronotal protuberances (p-pr) sclerotized, smooth; head, rostrum and pronotum darker than rest of body. Rostrum moderately slender. Pronotal protuberances fused at basis. Pronotum 2.2× as wide as long. Mesonotum distinctly smaller than metanotum. Urogomphi short, conical, with sclerotized apices. Abdominal segment VIII with rounded, prominent abdominal protuberance dorsally (Fig. 25A, B).

***Chaetotaxy*.** Sparse, setae short to medium, transparent. Head with one medium *os*. Rostrum with one *rs* (Fig. 25A). Pronotum with one elongate *as*, one *ls*, and four *pls* all almost equal in length. Dorsal parts of meso- and metathorax with two setae of various length, placed medially. Apices of femora with one medium-sized *fes* (Fig. 25A–C). Abdominal segments I–VIII with two medium-sized setae (one placed medially, the other laterally). Each lateral part of abdominal segments I–VIII with one medium-sized seta. Ventral parts of abdominal segments I–VIII with two medium-sized setae. Abdominal segment IX with two minute setae ventrally (Fig. 25A–C).

##### Biological notes.

The host plants of this species are several *Veronica* species: *V.anagallis-aquatica* L., *V.anagalloides* Guss., *V.beccabunga* L., *V.catenata* Pennell, *V.scutellata* L. (Kleine 1910; Urban 1930; Hustache 1931; Hoffmann 1958; Sprick 1997). The adults appear on the host plants in May. The females oviposit during June in the ovarial tissue, inducing a bulbous gall in which the larva develops.

##### Remarks and comparative notes.

This species is common in the whole of Europe and Anatolia. The adult is closely related to *G.miyoshii*, a vicariant species living in eastern Asia (Caldara 2008a; Alonso-Zarazaga et al. 2017). The immature stages confirm this relationship, as they share the postdorsal segment on the abdominal segments with one *pds* and the dorsal epicranium without *des_4_*.

### ﻿Key to the known mature larvae of *Gymnetron* species

The following key is based on the larvae of the five *Gymnetron* species described in this paper and one described by Jiang and Zhang (2015).

**Table:** 

1	Postdorsal segment on abdominal segments with one *pds*. Dorsal epicranium without *des_4_*. Postlabium with two *pms*	**2**
–	Postdorsal segment on abdominal segments with two *pds*. Dorsal epicranium with *des_4_*. Postlabium with three *pms*	**3**
2	Pronotum with six *prns* (as *prns* and *dpls*). *Des_1_* short; *des_2_* short; *des_3_* long, *fs_2_* and *fs_4_* short. Head with three *pes*	***G.miyoshii***
–	Pronotum with ten *prns*. *Des_1_* medium; *des_2_* absent; *des_3_* medium, *fs_2_* and *fs_4_* medium. Head with two *pes*	***G.villosulum***
3	Praedorsal segment on abdominal segments with two *pds*. Epicranium with *fs_3_*. Labral setae in one line	**4**
–	Praedorsal segment on abdominal segments with one *pds*. Epicranium without *fs_3_*. Labral setae in a triangle	**5**
4	Body cuticle covered with numerous reddish or brown asperities, black spots at base of setae. Head dark brown. Epipharynx with two *als* and three *ams*	***G.veronicae***
–	Body cuticle smooth, setae without black spots at base. Head pale yellow. Epiharynx with three *als* and two *ams*	***G.tibiellum***
5	Pronotum with seven setae. Meso- and metathorax with two *as* and one *ss*. Pedal area with three *pda. Mbs* present	***G.rotundicolle***
–	Pronotum with ten setae. Meso- and metathorax with one *as* and three *ss*. Pedal area with five *pda. Mbs* absent	***G.melanarium***

### ﻿Key to pupae of known *Gymnetron* species

The following key is based on the pupae of the five *Gymnetron* species described in this paper.

**Table:** 

1	Abdominal protuberance prominent, disc-shaped. Head with one *os*	**2**
–	Abdominal protuberance short, conical. Head with two *os*	***G.melanarium***
2	Pronotal protuberances with serrated margin. Rostrum without setae. Pronotum with one *ds* and two or three *pls*. Ventral parts of abdominal segment I–VII with three setae	**3**
–	Pronotal protuberances with smooth margin. Rostrum with one *rs*. Pronotum without *ds* and four *pls*. Ventral parts of abdominal segment I–VII with two setae	***G.villosulum***
3	Pronotum with three *pls*. Femora with two *fes*. Dorsal parts of abdominal segments I–VII with three or four setae	**4**
–	Pronotum with two *pls*. Femora with one *fes*. Dorsal parts of abdominal segments I–VII with two setae	***G.rotundicolle***
4	Pronotum with one *as*, and one *ls*. Meso- and metathorax with two setae. Dorsal parts of abdominal segments I–VII with three setae	***G.tibiellum***
–	Pronotum with two *as*, and without *ls*. Meso- and metathorax with three setae. Dorsal parts of abdominal segments I–VII with four setae	***G.veronicae***

## ﻿Discussion

### ﻿Comparison with immature stages of known Mecinini

It has been suggested that the number of palpomeres of the labial palpi is one of the most important morphological characters of larvae in the Mecinini (Skuhrovec et al. 2018). Phylogenetically, the basal state in weevils is the presence of two palpomeres on the labial palpi (Marvaldi 1997). In *Mecinus* there are species in the plesiomorphic state (e.g., *Mecinuscollaris* Germar, 1821; *Mecinusjanthinus* group), but also such with one palpomere (Gosik et al. 2020). All the *Gymnetron* species examined here have one labial palpomere, as do the few species of *Rhinusa* described to date. In contrast, *Cleopomiarus* and *Miarus* generally have two palpomeres, although in some *Cleopomiarus* species the basal palpomere is not distinctly separated from the labium and can appear to be just a single palpomere (Skuhrovec et al. 2018).

Another crucial generic-specific character in Mecinini larvae is the number of air tubes of the thoracic and abdominal spiracles. In *Gymnetron* all the spiracles are unicameral (Jiang and Zhang 2015). In the larvae of *Mecinus* species this character has two states: (1) all spiracles unicameral, as in *Gymnetron* and (2) the thoracic spiracle bicameral and the abdominal ones unicameral, as in some *Rhinusa* (Anderson 1973; May 1993; Ścibior and Łętowski 2018; Gosik et al. 2020). In contrast, all known larvae of *Cleopomiarus* and *Miarus* species have bicameral spiracles on the thorax and abdomen (Skuhrovec et al. 2018).

Another debatable state in the larvae is the number of epipharyngeal setae (especially *ams* and *mes*), which has not yet been completely resolved in Curculionidae (Gosik and Skuhrovec 2011; Stejskal et al. 2014; Trnka et al. 2015). In the Mecinini there are three *als*, two or three *ams*, and none or one *mes*. In our view, the final decision regarding the number of each seta is important, but not crucial, and the comparison between groups/genera should be made together for all three kinds of these epipharyngeal setae in order to make fewer errors when creating a differential diagnosis for the genera in the tribe.

The last important characteristic observed within the Mecinini tribe is the integument of the body covered with distinct asperities, both in the larval and pupal stages (Skuhrovec et al. 2018). This feature is very variable within each genus, probably owing to the distinctive environmental conditions within plant tissues.

With regard to the pupae, an uncommon character is the presence of two more or less sclerotized pronotal prominences, which can be smooth or serrated. Moreover, these pronotal protuberances (p-pr) are divisible into two parts with or without a stem from the pronotum and may have conical asperities or serrated margins. These prominences are present in all the *Gymnetron* species studied here, but also in some *Rhinusa* and a few *Mecinus* (Gosik 2010). The evolutionary significance of this character, which disappears altogether in the adult, is unclear.

### ﻿Differences between immatures at the species level

All the larvae and pupae of every species studied here, and also the three described by Jiang and Zhang (2015), have several characters distinguishing them from one another. These differences confirm that most of them belong to different groups, as suggested by the study of the adults (Caldara 2008a). Three species, very closely related on the basis of the adult morphology (*G.veronicae*, *G.tibiellum* and *G.auliense*), also have several characters in common in the larvae (presence of *fs_3_*; pro- and postdorsal folds of abdominal segments I–VI (VIII) with two *prs* and two *pds*; labral setae in one line) and in the pupae (sclerotized pronotal protuberances covered with conical asperities). The other two related species, *G.villosulum* and *G.miyoshii*, resemble each other more than the other species (in the larvae *des_4_* and *mbs* absent, postdorsal segment on abdominal segments with one *pds*; in the pupae pronotal protuberances smooth). The other species do not show clear relationships with each other or with the group of *G.veronicae* and *G.villosulum*. Only *G.vittipenne* could be related to the *G.villosulum* group, as also shown by the phylogenetic tree of the adults reported by Caldara (2008a).

### ﻿Biological and evolutionary considerations

This study confirms that all the Palaearctic species of the genus *Gymnetron* with known biologies live only on *Veronica*. No other species belonging to the Mecinini live on this genus of Plantaginaceae. All the species usually seem to feed on various species of this genus, partly unrelated to each other and belonging to different subgenera as currently considered (Albach et al. 2004). They feed on the ovary or the stem of the plant, sometimes forming more or less voluminous galls. A recent study of *Gymnetron* and *Rhinusa* indicated a strong phylogenetic signal with respect to host plants but a weaker one with respect to the particular plant structures occupied by the insects in question on different plant structures (Hernández-Vera et al. 2013).

## Supplementary Material

XML Treatment for Gymnetron

XML Treatment for Gymnetron tibiellum

XML Treatment for Gymnetron veronicae

XML Treatment for Gymnetron rotundicolle

XML Treatment for Gymnetron melanarium

XML Treatment for Gymnetron villosulum
